# Overcoming addition of phosphoethanolamine to lipid A mediated colistin resistance in *Acinetobacter baumannii* clinical isolates with colistin–sulbactam combination therapy

**DOI:** 10.1038/s41598-022-15386-1

**Published:** 2022-07-06

**Authors:** Sukrit Srisakul, Dhammika Leshan Wannigama, Paul G. Higgins, Cameron Hurst, Shuichi Abe, Parichart Hongsing, Thammakorn Saethang, Sirirat Luk-in, Tingting Liao, Naris Kueakulpattana, Aye Mya Sithu Shein, Lin Gan, Rosalyn Kupwiwat, Chanikan Tanasatitchai, Pattama Wapeesittipan, Phatthranit Phattharapornjaroen, Vishnu Nayak Badavath, Asada Leelahavanichkul, Tanittha Chatsuwan

**Affiliations:** 1 Department of Microbiology, Faculty of Medicine, Chulalongkorn University, King Chulalongkorn Memorial Hospital, Thai Red Cross Society, 1873 Rama 4 Road, Pathum Wan, Bangkok, 10330 Thailand; 2 Center of Excellence in Antimicrobial Resistance and Stewardship, Faculty of Medicine, Chulalongkorn University, Bangkok, Thailand; 3 Interdisciplinary Program of Medical Microbiology, Graduate School, Chulalongkorn University, Bangkok, Thailand; 4 School of Medicine, Faculty of Health and Medical Sciences, The University of Western Australia, Nedlands, WA Australia; 5 Institute for Medical Microbiology, Immunology and Hygiene, Faculty of Medicine and University Hospital Cologne, University of Cologne, Cologne, Germany; 6 German Centre for Infection Research, Partner Site Bonn-Cologne, Cologne, Germany; 7 Statistics, QIMR Berghofer Medical Research Institute, Brisbane, QLD Australia; 8 Department of Infectious Diseases and Infection Control, Yamagata Prefectural Central Hospital, Yamagata, Japan; 9 Department of Brain and Neurology, Mae Fah Luang University Hospital, Chiang Rai, Thailand; 10 School of Integrative Medicine, Mae Fah Luang University, Chiang Rai, Thailand; 11 Department of Computer Science, Faculty of Science, Kasetsart University, Bangkok, Thailand; 12 Department of Clinical Microbiology and Applied Technology, Faculty of Medical Technology, Mahidol University, Bangkok, Thailand; 13 Department of Physiology, Faculty of Medicine, Chulalongkorn University, Bangkok, Thailand; 14 Center of Excellence for Microcirculation, Faculty of Medicine, Chulalongkorn University, Bangkok, Thailand; 15 Department of General Surgery, Fuling Center Hospital of Chongqing City, Chongqing, China; 16 Chulabhorn International College of Medicine, Thammasat University, Bangkok, Thailand; 17 Department of Clinical Science, University of Bergen, Bergen, Norway; 18 Department of Emergency Medicine, Center of Excellence, Faculty of Medicine Ramathibodi Hospital, Mahidol University, Bangkok, Thailand; 19 Institute of Clinical Sciences, Department of Surgery, Sahlgrenska Academy, Gothenburg University, 40530 Gothenburg, Sweden; 20 School of Pharmacy & Technology Management,, SVKM’s Narsee Monjee Institute of Management Studies (NMIMS), Hyderabad, 509301 India; 21 Translational Research in Inflammation and Immunology Research Unit (TRIRU), Department of Microbiology, Chulalongkorn University, Bangkok, Thailand; 22 Pathogen Hunter’s Research Collaborative Team, Department of Infectious Diseases and Infection Control, Yamagata Prefectural Central Hospital, Yamagta, Japan

**Keywords:** Antimicrobials, Bacteria, Clinical microbiology, Drug discovery, Genetics, Microbiology, Molecular biology, Medical research, Molecular medicine

## Abstract

Overcoming colistin-resistant *Acinetobacter baumannii* (CoR-AB) has become a major concern due to the lack of effective antibiotics. This study aimed to explore the prevalence of CoR-AB clinical isolates in Thailand, their mechanisms of resistance, and test the efficacy of colistin plus sulbactam against CoR-AB isolates. The colistin resistance rate among carbapenem-resistant *A. baumannii* was 15.14%. The *mcr* gene or its variants were not detected in CoR-AB isolates by PCR screening. The lipid A mass spectra of CoR-AB isolates showed the additional [M–H]^−^ ion peak at *m*/*z* = 2034 that correlated to the phosphoethanolamine (pEtN) addition to lipid A (N = 27/30). The important amino acid substitutions were found at position S14P, A138T, A227V in PmrB that are associated with overexpression of the pEtN transferase (PmrC) and contributed the pEtN addition. The lipopolysacccharide production genes (*lpxACD*) were not related to lipid A mass spectra. A colistin plus sulbactam combination exhibited the synergy rate at 86.7% against CoR-AB isolates compare to sulbactam (85.89% resistance) or colistin (15.14% resistance) alone. The excellent synergistic activity of colistin plus sulbactam combination has the potential for the treatment of CoR-AB infections.

## Introduction

Colistin-resistant *Acinetobacter baumannii* (CoR-AB) strains have now been detected worldwide including Thailand, primarily due to the increasing use of colistin against carbapenem-resistant *A. baumannii* (CRAB)^1–4^. The prevalence of CoR-AB was recently reported to be 14.3% in Thailand^5^ and 13% in a recent global surveillance study^6^.

Colistin, a bactericidal antibiotic, binds to lipopolysaccharides (LPS) of the bacterial outer membrane of Gram-negative bacteria^7^. It was re-introduced as a “last-resort” antibiotic against carbapenem-resistant Gram negatives including *A. baumannii* as there were no therapeutic alternatives^8^. However, the incidence of CoR-AB has been increasingly reported over the recent years^6^. The mechanisms related to colistin resistance in *A. baumannii* have been investigated. The absence of LPS was shown to cause colistin resistance in *A. baumannii*. Moffatt, et al. found that the insertion inactivation or mutation in LPS biosynthetic genes (*lpxA*, *lpxC*, *lpxD*) lead to loss of LPS in CoR-AB isolates, but so far this has only been generated in laboratory conditions and not found in clinical isolates^9,10^. In clinical isolates, the major resistance mechanism is associated with the modification of LPS by phosphoethanolamine (pEtN) that reduces the binding affinity between colistin and lipid A^11^. The pEtN modification is mediated by the overexpression of PmrC (pEtN transferase)^12^ which might result from the mutations in PmrAB two-component system^13^, a bacterial receptor for divalent cations and polymyxin antibiotics^14^. Moreover, the mobile colistin resistant (*mcr*) genes and variants, that encoded pEtN transferase, have been described in CoR-AB isolates, however this is still a relatively rare mechanism in *A. baumannii*^15,16^.

Several antibiotic combinations have been proposed as therapeutic choices to treat CoR-AB. Such as colistin plus rifampicin or vancomycin were found more synergistic and effective against CoR-AB clinical isolates than other combinations^17,18^. However, the colistin plus rifampicin combination did not show a significant clinical response when compared with colistin monotherapy^19^. A combination of colistin plus sulbactam or fosfomycin were investigated against CRAB and exhibited synergistic activity^20–22^ and considered to use with colistin^23,24^.

This study aimed to investigate the prevalence of colistin resistance and their mechanisms of resistance in *A. baumannii* clinical isolates from Thailand, and to explore the synergistic activity of colistin plus sulbactam (COL/SUL), colistin plus fosfomycin (COL/FOS), and sulbactam plus fosfomycin (SUL/FOS) combinations against CoR-AB clinical isolates.

## Results

### The increasing prevalence of CoR-AB in CRAB in tertiary care hospital of Thailand

*A. baumannii* clinical isolates (n = 317) were collected from different patients at King Chulalongkorn Memorial Hospital, Bangkok, Thailand between 2017 and 2019. All of the isolates were confirmed as *A. baumannii* by *gyrB* multiplex PCR. All CRAB isolates included in this study were resistant to both imipenem and meropenem. The rates of resistance to amikacin, ciprofloxacin, fosfomycin, levofloxacin, and sulbactam were 81.7%, 98.74%, 80.44%, 94.95%, and 85.89% respectively (Table 1). The colistin resistance rate was 15.14%.

**Table 1:** Antimicrobial susceptibility of *A. baumannii* clinical isolates (n = 317).

Antimicrobial agent	MIC range (mg/L)	MIC_50_ (mg/L)	MIC_90_ (mg/L)	% Resistance
COL	0.125–64	1	4	15.14
MEM	32– > 256	64	128	100
IMP	16– > 256	128	256	100
AMK	1– > 256	> 256	> 256	81.7
CIP	0.5– > 256	64	256	98.74
LVX	0.5–128	16	32	94.95
FOS^a^	64– > 256	256	> 256	80.44
SUL^b^	1– > 256	32	64	85.89

*AMK* amikacin, *CIP* ciprofloxacin, *COL* colistin, *FOS* fosfomycin, *IMP* imipenem, LVX levofloxacin, *MEM* meropenem, *MIC* minimum inhibitory concentration, *SUL* sulbactam.

^a^The interpretation criteria for Enterobacteriaceae by the CLSI was used for fosfomycin susceptibility.

^b^The interpretation criteria of ampicillin/sulbactam for *Acinetobacter* spp. by the CLSI was used for sulbactam susceptibility.

### PmrCAB and LpxD substitutions were associated with colistin resistance

**Figure 1:**
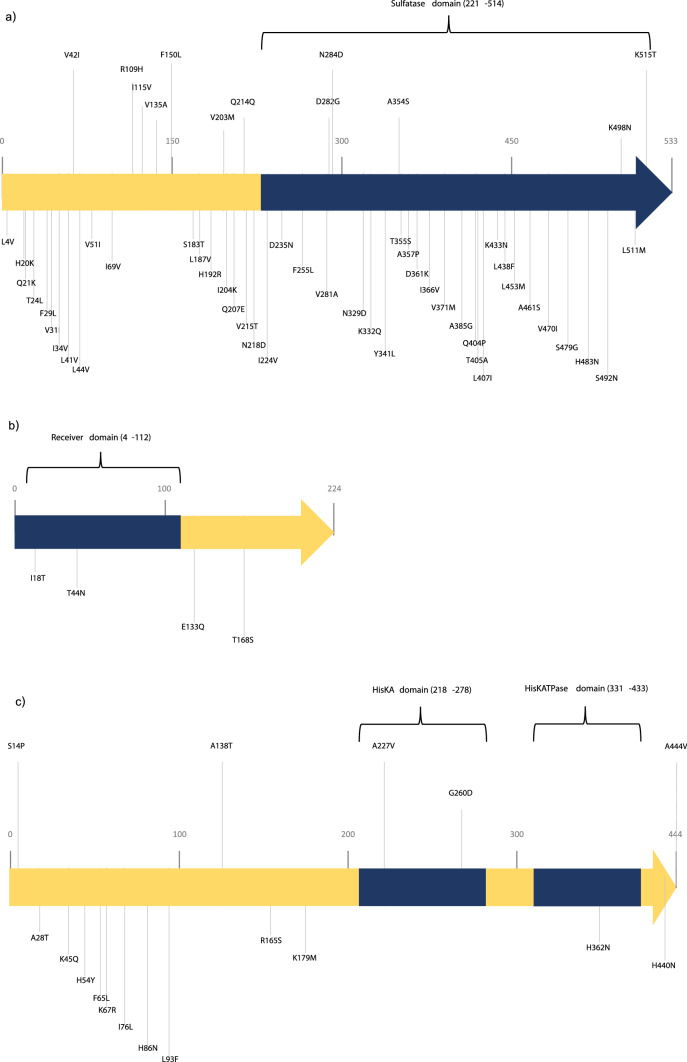
The amino acid substitution in PmrC (**a**), PmrA (**b**), and PmrB (**c**) amino acid sequences of colistin-resistant *A. baumannii* (CoR-AB) clinical isolates (n = 30).

The two-component system (TCS), PmrA and PmrB, is a responder to external stimuli including divalent cations and polymyxin antibiotics^14^. The PmrAB TCS regulates expression of *pmrC* that encodes pEtN transferase which can mediate resistance to colistin in *A. baumannii*^12^. The 30 CoR-AB isolates were selected for investigation of mutations in the *pmrCAB* operon. The comparison between the amino acid sequence of CoR-AB and that of *A. baumannii* ATCC 19606 and ATCC 17978 revealed amino acid substitutions in 22 (73.3%) CoR-AB isolates (Fig. 1). These substitutions were found in 48 positions in PmrC (Fig. 1a, 4 positions in PmrA (Fig. 1b), and 20 positions in PmrB (Fig. 1c). For PmrC, the N284D substitution was found in all isolates. The I18T and T44N substitutions in PmrA were found in 22 isolates (Fig. 1b). Eight isolates harbored unique and different amino acid mutations in PmrC and PmrB with no mutation in PmrA. Of 8 isolates, 13 substitutions were detected in PmrC including V42I, R109H, I155V, V135A, F150L, V203M, R214Q, D282G, V321I, A354S, V470I, K498N, and K515T. In addition, PmrB of these isolates contained 7 different amino acid substitutions including S14P, A138T, R165P, G260D, L274F, H440N, and A444V.

We also looked for inactivation of LPS biosynthesis that is associated with the *lpxACD* by comparing the amino acid sequence with *A. baumannii* ATCC 17978 and found 28 isolates with the amino acid substitution at E117K in LpxD protein. The other 2 isolates harbored mutations at V3A in LpxD. Amino acid substitutions in LpxA and LpxC were not detected in CoR-AB isolates in this study.

This study also screening the *mcr* gene and its variants using the 2 set of multiplex PCR. However, the representative CoR-AB isolates (n = 30) were not detected of any *mcr* gene and variants.

### Colistin resistance in *A. baumannii* is associated with pEtN modification

**Figure 2:**
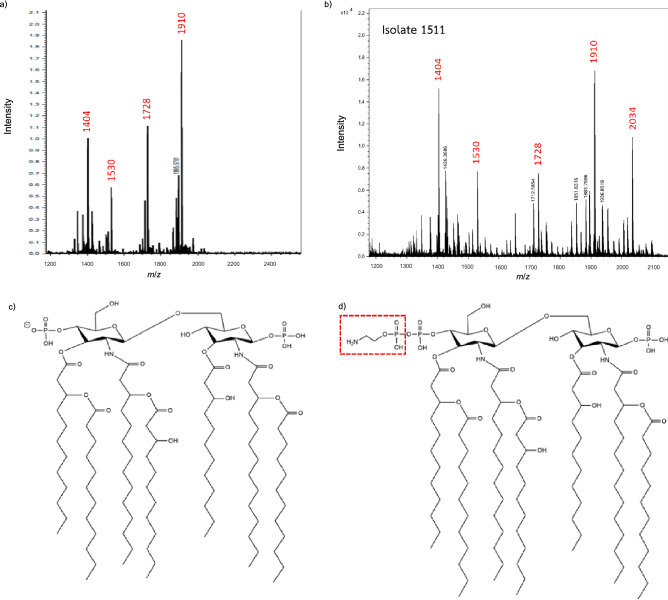
The lipid A spectrum of *A. baumannii* ATCC 19606 (**a**) and colistin-resistant *A. baumannii* (**b**). The colistin-resistant isolate was detected additional [M–H]^−^ peak at *m*/*z* 2034 that associated with phosphoethanolamine addition to hepta-acylated lipid A (*m*/*z* 1910). The predicted structure of hepta-acylated lipid A (**c**) and hepta-acylated lipid A with phosphoethanolamine addition (**d**).

The addition of pEtN is known to be involved in colistin resistance of *A. baumannii*^11^. We therefore analyzed the lipid A profile byMALDI-TOF MS of CoR-AB isolates (n = 30) compared with that of *A. baumannii* ATCC 19606. The lipid A spectrum of *A. baumannii* ATCC 19606, representing a colistin-susceptible isolate, showed 4 major peaks which were consistent with *bis*-phosphoryl hepta-acylate lipid A (*m*/*z* 1910), *bis*-phosphoryl hexa-acylate lipid A (*m*/*z* 1728), *bis*-phosphoryl penta-acylate lipid A (*m*/*z* 1530), and *bis*-phosphoryl tetra-acylate lipid A (*m*/*z* 1404) (Fig. 2a). In the CoR-AB isolates (n = 30), all isolates contained the predominant peaks at *m*/*z* 1404 and 1910. In twenty-seven isolates, an additional peak at *m*/*z* 2034, corresponding to the addition of pEtN (predicted *m*/*z* 124) to *bis*-phosphoryl hepta-acylate lipid A, was found (Fig. 2b–d and Supplementary Fig. 1). The isolate 176, 216, and 1126 did not have the additional peak at *m*/*z* 2034 (Supplementary Fig. 2).

### Checkerboard assay confirmed the high synergistic rate of COL/SUL combination

The COL/SUL, COL/FOS, and SUL/FOS combinations were tested for synergistic activity against the 30 CoR-AB isolates. Among the three combinations, the most effective was COL/SUL (86.7% synergy), followed by SUL/FOS (70% synergy), and COL/FOS (33.3% synergy) (Table 2). The partial synergy observed was 53.3%, 26.7%, and 10% in COL/FOS, SUL/FOS, and COL/SUL combinations, respectively. Four isolates showed an indifferent effect in COL/FOS combination. The isolate 213 and A5 had indifferent effects with the SUL/FOS and COL/SUL combinations. There were no antagonistic effects observed.

**Table 2:** Result of checkerboard synergy test of three antibiotic combinations against 30 colistin-resistant *A. baumannii* clinical isolates.

Antibiotic combination	Isolate(s) with the indicated test result (isolates/%)		
	Synergy (FICI ≤ 0.5)	Partial synergy (0.5 < FICI < 1)	Indifferent (1 ≤ FICI < 4)
Colistin + Sulbactam	26/86.7%	3/10%	1/3.3%
Colistin + Fosfomycin	10/33.33%	16/53.3%	4/13.3%
Sulbactam + Fosfomycin	21/70%	8/26.7%	1/3.3%

### in vitro time-kill assay confirmed the COL/SUL combination as a possible therapeutic option against CoR-AB

**Figure 3:**
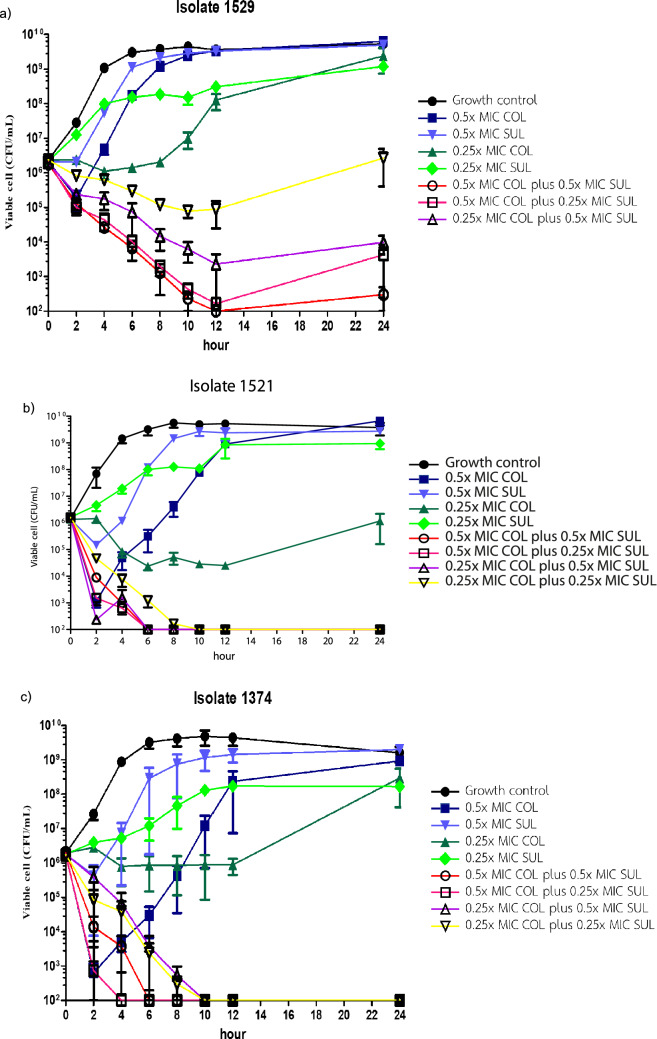
in vitro time-kill study colistin-resistant *A. baumannii* isolates (**a**) 1529, (**b**) 1521, (**c**) 1374.

To confirm the synergistic effect of the antibiotic combinations, six representative CoR-AB isolates were selected to determine the efficacy of COL/SUL combination by time-kill assay. This study used the combinations between 0.5X MIC or 0.25X MIC of colistin with 0.5X MIC or 0.25X MIC of sulbactam to test the synergistic activity (Fig. 3). All of the combinations expressed the synergy effect between colistin and sulbactam in all tested isolates. Every combination showed excellent bactericidal activity when tested against isolate 1251, 1341, 1374, and 1521. The combination of 0.5X MIC of colistin with 0.25X MIC of sulbactam, tested against isolate 1529, showed re-growth of bacteria at 24 h. The combination of 0.25X MIC of colistin with 0.5X MIC of sulbactam was bacteriostatic as opposed to bactericidal in isolate 1529. The combination of 0.25X MIC of colistin with 0.25X MIC of sulbactam showed no bactericidal activity against isolate 1129 and 1529.

### The COL/SUL combination eradicated the CoR-AB infection in in vivo mouse models

**Figure 4:**
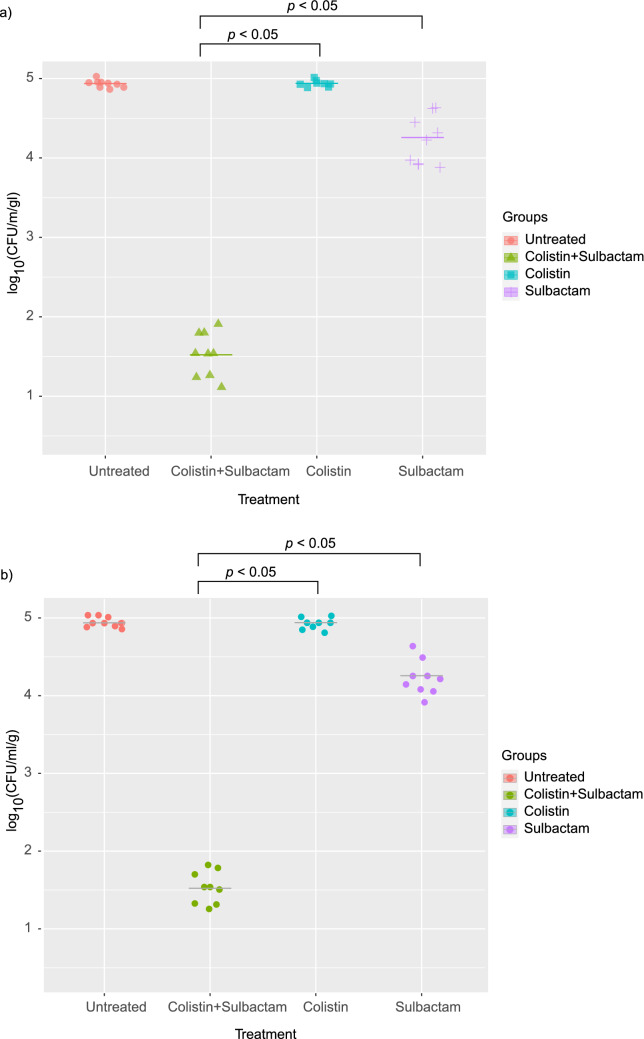
Colistin and sulbactam combination therapy is efficacious in mouse thigh models of infection. (**a**) Single-dose treatment at 1 h post infection of either colistin (20 mg kg^−1^, i.p. n = 10), sulbactam (120 mg kg^−1^, p.o. n = 10), untreated (n = 10), or the combination (n = 10) in a neutropenic mouse thigh infection model using representative colistin-resistant *A. baumannii* isolates (**a**) 1251 and (**b**) 1374. Colony-forming units (CFU) within thigh tissue were enumerated at 8 h post infection and compared to the untreated group. Horizontal lines represent geometric mean of the bacterial load for each treatment group. *P* values were determined using a two-sided, Mann–Whitney U-test.

**Figure 5:**
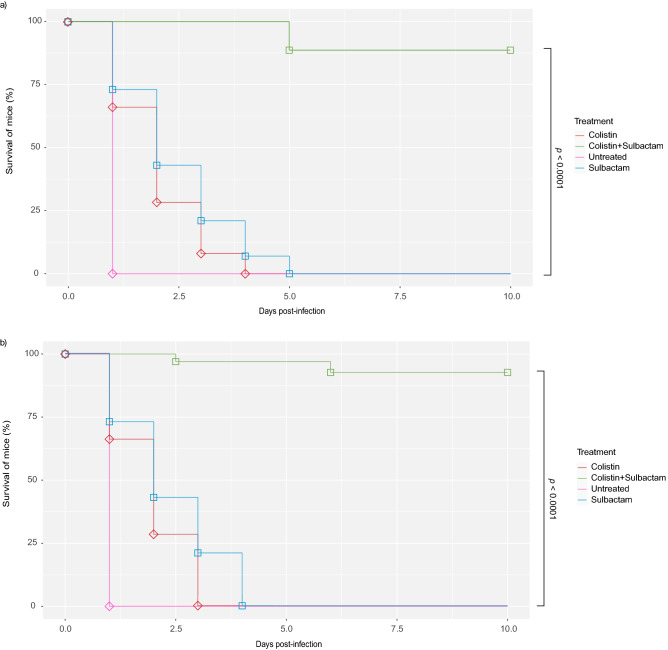
Colistin and sulbactam combination therapy is efficacious in Mouse bacteremia model. Survival curve of representative colistin-resistant *A. baumannii* isolates (**a**) 1251 and (**b**) 1374 bacteremia infection dosed at 1, 24, 48, 72, 96, and 120 h post infection as outlined above for either colistin (20 mg kg^−1^, i.p. n = 10), sulbactam (120 mg kg^−1^, p.o. n = 10), untreated (n = 10), or the combination (n = 10). *P* values were determined using a two-sided, Mann–Whitney U-test.

To confirm the synergistic activity of COL/SUL, the two mouse models were treated with the COL/SUL combination against representative CoR-AB (isolates 1251 and 1374) infections. The mice were infected with 1 × 10^6^ CFU/mL of bacteria in the thigh or peritoneal cavity of C57BL/6 mice then treated with colistin or sulbactam alone, or COL/SUL combination (Colistin 20 mg kg^−1^, and sulbactam 120 mg kg^−1^). The bacterial count of combination-treating group was significantly lower than monotherapy group in the thigh infection mouse model (*p* < 0.05) (Fig. 4). The COL/SUL combination exhibited significantly superior survival rate in the peritoneal infection model compared to monotherapy groups (*p* < 0.0001) (Fig. 5).

## Discussion

The rise of CoR-AB isolates has led to few therapeutic options to treat this pathogen, and an increased mortality rate^2^. This study has revealed the prevalence and mechanisms of colistin resistance in carbapenem-resistant *A. baumannii* clinical isolates from Thailand. The results showed that the prevalence of CoR-AB has risen to 15.4%, an increase from 3.6 to 9.3% recorded in 2014–2015 in Thailand^25,26^. Moreover, the rate of CoR-AB was higher than that in a worldwide surveillance study (4.1% in 2001–2016)^6^.

The modification of lipid A by pEtN associated with the mutations of *pmrCAB* operon was the major mechanism of colistin resistance in CoR-AB isolates in this study. The lipid A analysis by MALDI-TOF MS in negative mode revealed that the lipid A of *A. baumannii* consisted of the [M–H]^−^ ion peaks at *m*/*z* 1404, 1530, 1728, and 1910. These ion peaks were reported as the representative of tetra-acylated, penta-acylated, hexa-acylated, hepta-acylated lipid A, respectively^27^. Moreover, the [M–H]^−^ ion peaks at *m*/*z* 2034 was found in the majority of CoR-AB isolates (n = 27). Previous studies described that the peak at *m*/*z* 2034 corresponded to the hepta-acylated lipid A with pEtN addition and related to the reduced colistin susceptibility in CoR-AB isolates^11^. The addition of pEtN was presumably related to the mutation in *pmrCAB* operon^13^. The identical amino acid substitutions were found in multiple positions on PmrCAB of CoR-AB clinical isolates (n = 22). Gerson, et al. indicated that the multiple substitutions hypothetically associated with the homologous recombination between *pmrCAB* of *A. baumannii* and the different clonal lineage^28^. Importantly, this study found the amino acid substitutions at position A227V on histidine kinase (HisKA) domain of PmrB and S14P and A138T on transmembrane domain of PmrB. This substitution in the HisKA domain has been described in the addition of pEtN to lipid A and colistin resistance^29,30^. In addition, both substitutions on the transmembrane domain were predictably correlated to colistin resistance^12,31^. The plasmid-containing *mcr* gene has been shown to induce colistin resistance in other Gram-negative bacteria^32^. However, the *mcr* gene and its variants are rarely found in *A. baumannii* and were not detected in our CoR-AB isolates. Unexpectedly, the predicted peak of pEtN addition was absent in 3 isolates of CoR-AB. The colistin resistance in these isolates may be associated to other mechanisms such as the overexpression of efflux pumps^33^ or the expression of heteroresistant phenotype^34^. Furthermore, the mutational or insertion inactivation of *lpxA*, *lpxC*, and *lpxD* genes affected to the synthesis of LPS and possibly related to colistin resistance in *A. baumannii*^9^. However, we observed substitutions at V3A and E117K in LpxD, but this result was not related to the lipid A spectra seen in MALDI-TOF MS.

An antibiotic combination is one strategy to solve the lack of effective antibiotics against CoR-AB^2^. This study confirmed the synergistic activity of COL/SUL, COL/FOS, and SUL/FOS combinations in vitro, including checkerboard assay and time-kill assay, and in vivo infection models. The COL/SUL combination expressed superior synergy rate (86.7%) than SUL/FOS (70%) and COL/FOS (33.3%) combinations in a checkerboard assay. The colistin-based combination with rifampicin or vancomycin showed successful in vitro synergistic results in previous studies^17^ and combination of colistin with rifampicin or tigecycline further exhibited the efficacy to restrain the colistin heteroresistant sub-population at low concentrations^35^. However, the clinical data of colistin plus rifampicin treatment compared to colistin monotherapy did not show a significant difference in clinical response^19^. The synergy rate of the COL/SUL combination in this present study was greater than that of a previous study (50% synergy rate) which showed colistin plus vancomycin was most effective (90% synergy rate)^18^. This is the first report of in vitro synergistic activity of COL/FOS and SUL/FOS combinations against CoR-AB clinical isolates. Despite the synergy rate, SUL/FOS was inferior to COL/SUL but it might be considered as an alternative treatment of CoR-AB infections.

The COL/SUL combination was selected by its greatest synergy rate to confirm the synergistic activity by time-kill assay and in vivo mouse model. The time-kill assay of COL/SUL affirmed the synergy effect in all tested CoR-AB isolates and showed rapid bactericidal activity. The synergistic activity of COL/SUL was observed in the mouse model. This combination eliminated the CoR-AB both in the thigh infection and systemic infection models. We hereby presented the first confirmative data that the COL/SUL combination could be an option to treat CoR-AB infections.

This study had some limitations. The association between the expression of PmrCAB with the level of colistin resistance and the other colistin resistant mechanisms such as the activation of *eptA* gene or the expression of antibiotic efflux pumps were not investigated. Further studies are planned to examine these gaps and their effect on the killing activity of COL/SUL combination. These current data suggested that the COL/SUL combination has potential to treat against CoR-AB infections. However, further studies should be performed to investigate the microbiology outcome and patient response in a clinical trial.

In conclusion, our study showed the rapid overcoming of CoR-AB isolates in Thailand. The colistin resistance in these CoR-AB isolates was related to the addition of pEtN to lipid A in the outer membrane. The mutations in *pmrCAB* operon were found at the functional positions that were predictably related to the pEtN modification. The COL/SUL combination was investigated in vitro and in vivo synergistic activity and expressed the highest synergy rate compared to the other combinations tested. The synergistic activity of this combination in time-kill and mouse models exhibited significant synergy effect against CoR-AB clinical isolates. These data suggest that the COL/SUL combination should be considered as a treatment option of CoR-AB infections.

## Materials and methods

### Bacterial isolates and antimicrobial susceptibility testing

The 317 *A. baumannnii* clinical isolates were obtained from King Chulalongkorn Memorial Hospital during 2017 to 2019. Isolates were identified as *A. baumannii* by multiplex *gyrB* multiplex PCR^36^. The colistin susceptibility was determined by broth microdilution. Susceptibility to other antibiotics including meropenem, imipenem, amikacin, ciprofloxacin, levofloxacin, sulbactam, and fosfomycin were determined by agar dilution method. The reference strains were used as described in Clinical and Laboratory Standard Institute (CLSI) guideline^37^. The susceptibility criterion of colistin, meropenem, imipenem, amikacin, ciprofloxacin, and levofloxacin were interpreted according to CLSI guidelines. In addition, the susceptibility criteria of ampicillin/sulbactam of *Acinetobacter spp*. was applied for sulbactam susceptibility. For fosfomycin susceptibility, the glucose-6-phosphate (G6P) was used as supplement and the interpretation criteria for Enterobacterales was applied.

### Sequencing of *pmrCAB*, *lpxA*, *lpxC*, and *lpxD*

The genomic DNA of CoR-AB isolates were extracted by Purelink genomic DNA mini kit (Invitrogen). The primers and PCR conditions were applied with modification from previous studied^9,12^. The amplicons were purified with Hiyield Gel/PCR DNA mini kit (RBCscience). The purified amplicons were sequenced by using the Bigdye Terminator V3.1 Cycler sequencing kit. Sequences were determined and analyzed by comparing with nucleotide sequence of *A. baumannii* ATCC 17978 (Genbank accession number CP000521.1) and *A. baumannii* ATCC 19606 (Genbank accession number CP045110.1).

### Screening of *mcr* genes

The specific primers of *mcr-1* to *mcr-9* that described in previous studies^38,39^ were used in this study. Two multiplex PCR were performed for detection of *mcr-1* to *mcr-5* and *mcr-6* to *mcr-9.* The amplicons were detected by gel electrophoresis with 2% agarose gel.

### Analysis of lipid A structure

The lipid A of CoR-AB isolates were extracted and purified by using ammonium hydroxide-isobutyric acid method as previously described^11^. In brief, the bacteria were grown in LB broth at 37 °C with shaking overnight, the cell pellet was collected by centrifugation and lyophilized. Lyophilized pellets (20 mg) were resuspended in 800 µL of concentrated isobutyric acid:1 M ammonium hydroxide (5:3 v/v) in 1.5-mL tube and incubated at 100 °C for 1.5–2 h with occasional vortexing. Samples were then cooled on ice and centrifuged at 2000×*g* for 15 min. Supernatants were transfer to new tubes and added with equal volumes of water, then lyophilized. After that, samples were washed twice with 400 µL of methanol then the insoluble lipid A pellets were immediately dissolved in 100 to 200 µL of chloroform:methanol:water (12:6:1 v/v/v) before MALDI-TOF analysis. The lipid A structure was analyzed by JMS-S3000 SpiralTOF-plus MALDI-TOF MS (JOEL Ltd., Japan) in negative spiral mode. A 1 µL aliquot of each sample was spotted on the halide-targeted plate and covered with 1 µL of the 10 mg/mL norharmane (as matrix) in the same solvent as the sample. Each spectrum was collected with a laser shot with 45% laser intensity. An ESI tuning mix was used to calibrate MALDI-TOF MS. Further calibration was performed using lipid A extracted from *E. coli* ATCC 25922.

### Synergistic activity testing by checkerboard assay

The combination of colistin plus sulbactam (COL/SUL), colistin plus fosfomycin (COL/FOS), and sulbactam plus fosfomycin (SUL/FOS) were tested to determine their synergistic activity against CoR-AB isolates. Checkerboard assay was performed in 96-well plate by mixing the different concentrations of two antibiotics in combinations. In brief, the two-fold serial dilution of antibiotic A was prepared in 96-well plate then adding the serial dilution of drug B in different panel. After mixing antibiotic properly, the 20 µL of a 10^6^ CFU/mL of bacterial suspension was added to each well. The plates were incubated at 37 °C for 18–24 h. Every combination was tested in duplicate. Synergistic activity was determined by fractional inhibitory concentration index (FICI), calculated by the sum of the FIC of each antibiotic. The FIC was defined as the MIC of antibiotic in combination divided by the MIC of antibiotic when tested alone. Synergy is defined as a FICI < 0.5, partial synergism as a FICI between 0.5 and 1, indifference as a FICI of > 1 but ≤ 4, and antagonism as a FICI of > 4^22^.

### In vitro time-kill assay

Time-kill assay was performed for COL/SUL combination against representative CoR-AB isolates. Flasks containing an antibiotic combination and antibiotic alone were inoculated with 10^6^ CFU/mL of CoR-AB isolates in a final volume of 10 mL and incubated at 37 °C with shaking. The concentrations of 0.5X MIC and 0.25X MIC of each antibiotic were used in this study. Aliquots were collected at time 0, 2, 4, 6, 8, 10, 12, and 24 h after incubation, then serially diluted in sterile saline for determination of viable cell counts. Diluted samples (10 µL) were spotted on MHA with 10 spots then incubated at 37 °C for 18–24 h. The colonies were counted and calculated to CFU/mL. All these experiments were performed in triplicate. The mean and standard deviation of viable bacterial cells in each condition were plotted on a semi-log graph. Bactericidal activity was determined as 3log_10_ CFU/mL reduction in colony count relative to the initial inoculum. Synergy is defined as a ≥ 2 log_10_ CFU/mL decrease when compared with the most single active antibiotic^22^.

### Animal studies

All animal studies were conducted according to guidelines and protocols approved by the Institutional Animal Care and Use Committee of the Faculty of Medicine, Chulalongkorn University, Bangkok, Thailand, based on the National Institutes of Health (NIH), USA. All animal studies were performed with male, 8-week-old C57BL/6 mice purchased from Nomura Siam International (Pathumwan, Bangkok, Thailand). Male mice were used in accordance with previously established models as well as ease of housing and randomization^40,41^. Sample size was selected based on the results of a preliminary infection trial. Before infection, mice were relocated at random from a housing cage to treatment or control cages. No animals were excluded from analyses and blinding was considered unnecessary.

### Mouse thigh infection model

The combination of colistin and sulbactam was tested against representative CoR-AB isolates (1251 and 1374) in a neutropenic mouse thigh infection model as described previously^40^. Male C57BL/6 mice were rendered neutropenic by cyclophosphamide, dosed at 150 and 100 mg kg^−1^ delivered on days -4 and -1 prior to infection. Bacteria were suspended in sterile saline and adjusted to a concentration of ~ 1 × 10^6^ CFU per infection site and injected into the right and left thighs of five mice per treatment group. At 1 h post infection, mice received either colistin (20 mg kg^−1^, i.p. n = 10), sulbactam (120 mg kg^−1^, p.o. n = 10), untreated (n = 10), or the combination (n = 10). Mice were euthanized 8 h post-infection, and the thigh tissue was aseptically collected, weighed, homogenized, serially diluted in PBS and plated onto solid LB (50 µg mL^−1^). Plates were incubated overnight at 37 °C and colonies were quantified to determine bacterial load.

### Mouse bacteraemia model

The combination of colistin and sulbactam was tested against representative CoR-AB isolates (1251 and 1374) in an immunocompetent bacteraemia infection model as described previously^40^. Male C57BL/6 mice were infected intraperitoneally with ~ 1 × 10^6^ CFU of bacteria with 5% porcine mucin (Sigma-Aldrich). Infections were allowed to establish for 1 h prior to treatment with colistin, sulbactam, or the combination. With the encouraging reduction in CFU observed in the thigh infection model, dosing was administered as described above. Clinical endpoint was determined using a five-point body condition score analysing weight loss, decrease in body temperature, respiratory distress, hampered mobility, and hunched posture. Experimental endpoint was defined as 10 days post infection for mice not reaching clinical endpoint.

### Data analysis

All statistical analysis was conducted using R statistic package^42^. The data were compared by either unpaired two-tailed Student’s t-test or unpaired two-tailed Mann–Whitney’s U test. Statistical significance was accepted at *p* < 0.05, *p* < 0.01, *p* < 0.001, and *p* < 0.0001.

### Ethics approval

The study protocol was approved by the Institutional Review Board (IRB) of the Faculty of Medicine, Chulalongkorn University, Bangkok, Thailand (COA No. 045/2019, IRB No. 315/62) was performed in accordance with the ethical standards as laid down in the 1964 Declaration of Helsinki and its later amendments and comparable ethical standards. Animal care and use protocol are based upon the National Institutes of Health (NIH), USA. The protocol was approved by the Institutional Animal Care and Use Committee of the Faculty of Medicine, Chulalongkorn University, Bangkok, Thailand (Certificate No- 033/2563, Research Project No. 020/2563). The study was carried out in compliance with the ARRIVE guidelines (Animal Research: Reporting of In Vivo Experiments).

### Informed consent

For this retrospective study of anonymous clinical isolates, the requirement for informed consent from patients was waived by Institutional Review Board (IRB) of the Faculty of Medicine, Chulalongkorn University, Bangkok, Thailand (COA No. 045/2019, IRB No. 315/62).

## Supplementary Information

Supplementary Information.

## Data Availability

The authors confirm that the data supporting the findings of this study are available within the article.
