# Supplementary material for: Overcoming addition of phosphoethanolamine to lipid A mediated colistin resistance in Acinetobacter baumannii clinical isolates with colistin–sulbactam combination therapy
Source: Sci Rep. 2022 Jul 6;12:11390. doi: 10.1038/s41598-022-15386-1 (PMC9259700; doi:10.1038/s41598-022-15386-1)
Supplement: Supplementary file 1 — Supplementary Information. [file 41598_2022_15386_MOESM1_ESM.docx]

**Overcoming addition of phosphoethanolamine to lipid A mediated colistin resistance in *Acinetobacter baumannii* clinical isolates with colistin-sulbactam combination therapy**

Sukrit Srisakul^1,2,†^, Dhammika Leshan Wannigama^1,2,4, #^, Paul G. Higgins^5,6, †^,Cameron Hurst^7 , †^, Shuichi Abe^8^ , Parichart Hongsing^9,10, †^, Thammakorn Saethang^11^, Sirirat Luk-in^12, †^, Tingting Liao ^13,14^, Naris Kueakulpattana ^1,2^, Aye Mya Sithu Shein^1,2,3^, Lin Gan^15^, Rosalyn Kupwiwat^16^, Chanikan Tanasatitchai^1,2^, Pattama Wapeesittipan^17^, Phatthranit Phattharapornjaroen^18,19^, Vishnu Nayak Badavath^20^, Asada Leelahavanichkul^1,21^, Tanittha Chatsuwan^1,2,#,*^

^1^ Department of Microbiology, Faculty of Medicine, Chulalongkorn University, King Chulalongkorn Memorial Hospital, Thai Red Cross Society, Bangkok, Thailand.

^2^ Antimicrobial Resistance and Stewardship Research Unit, Faculty of Medicine, Chulalongkorn University, Bangkok, Thailand.

^3^​Interdisciplinary Program of Medical Microbiology, Graduate School, Chulalongkorn University, Bangkok, Thailand.

^4^ School of Medicine, Faculty of Health and Medical Sciences, The University of Western Australia, Nedlands, Western Australia, Australia.

^5^ Institute for Medical Microbiology, Immunology and Hygiene, Faculty of Medicine and University Hospital Cologne, University of Cologne, Cologne, Germany.

^6^ German Centre for Infection Research, Partner site Bonn-Cologne, Cologne, Germany.

^7^ Statistics, QIMR Berghofer Medical Research Institute, Brisbane, Queensland, Australia.

^8^ Department of Infectious Diseases and Infection Control, Yamagata Prefectural Central Hospital, Yamagata, Japan.

^9^ Mae Fah Luang University Hospital, Chiang Rai, Thailand.

^10^ School of Integrative Medicine, Mae Fah Luang University, Chiang Rai, Thailand.

^11^ Department of Computer Science, Faculty of Science, Kasetsart University, Bangkok, Thailand.

^12^ Department of Clinical Microbiology and Applied Technology, Faculty of Medical Technology, Mahidol University, Bangkok, Thailand.

^13^ Department of Physiology, Faculty of Medicine, Chulalongkorn University, Bangkok, Thailand.

^14^ Center of Excellence for Microcirculation, Faculty of Medicine, Chulalongkorn University

^15^ Department of General surgery, Fuling Center Hospital of Chongqing City, Chongqing, China

^16^ Chulabhorn International College of Medicine, Thammasat University, Thammasat University ^17^Department of clinical science, University of Bergen, Norwayospital, Bangkok, Thailand.

^18^ Department of Emergency Medicine, Center of Excellence, Faculty of Medicine Ramathibodi Hospital, Mahidol University, Bangkok, Thailand

^19^ Institute of Clinical Sciences, Department of Surgery, Sahlgrenska Academy, Gothenburg University, 40530 Gothenburg, Sweden

^20^ Chitkara College of Pharmacy, Chitkara University, Punjab, 140401, India

^21^ Translational Research in Inflammation and Immunology Research Unit (TRIRU), Department of Microbiology, Chulalongkorn University, Bangkok, Thailand

^†^These authors contributed equally to this work

^#^These authors jointly supervised this work

*Corresponding Author: Tanittha Chatsuwan, PhD

**Supplementary data**

Supplementary
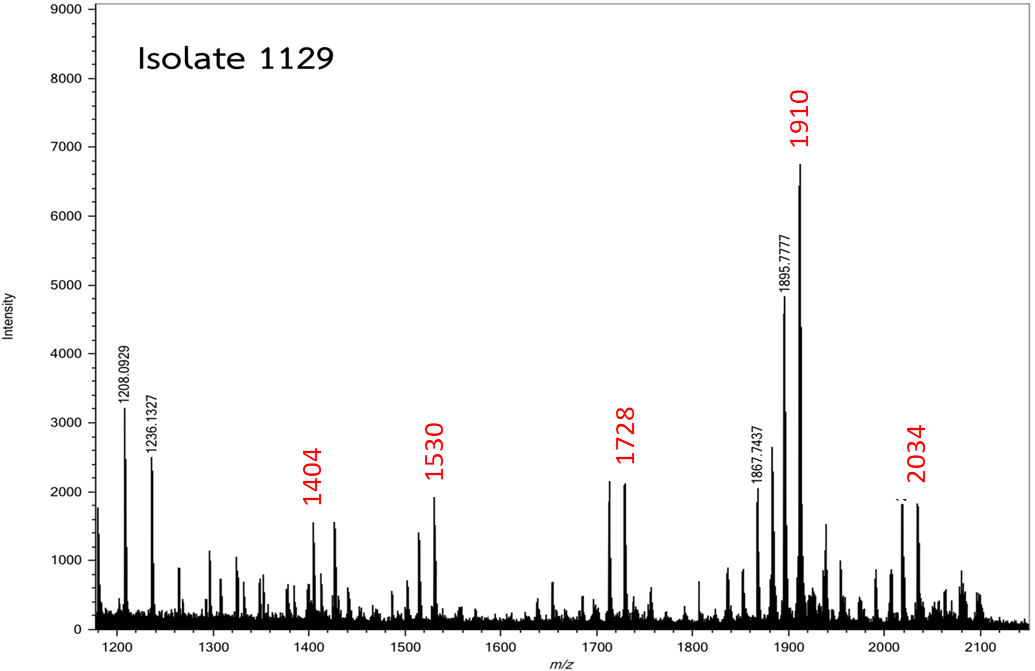

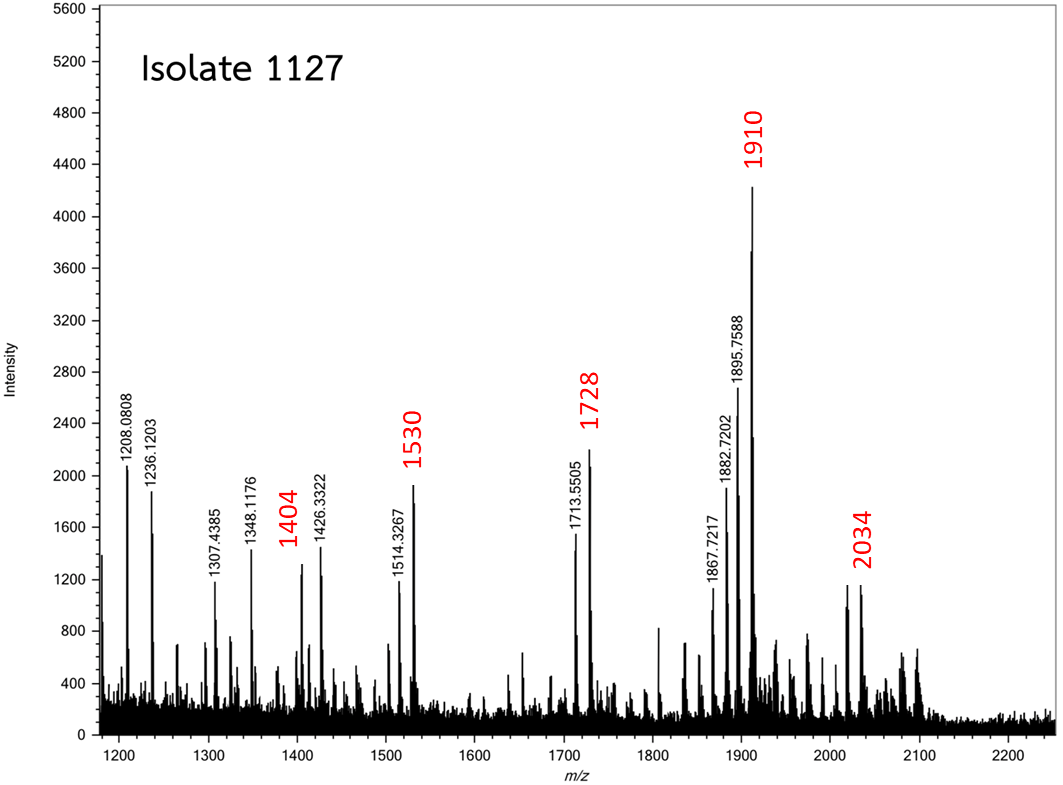

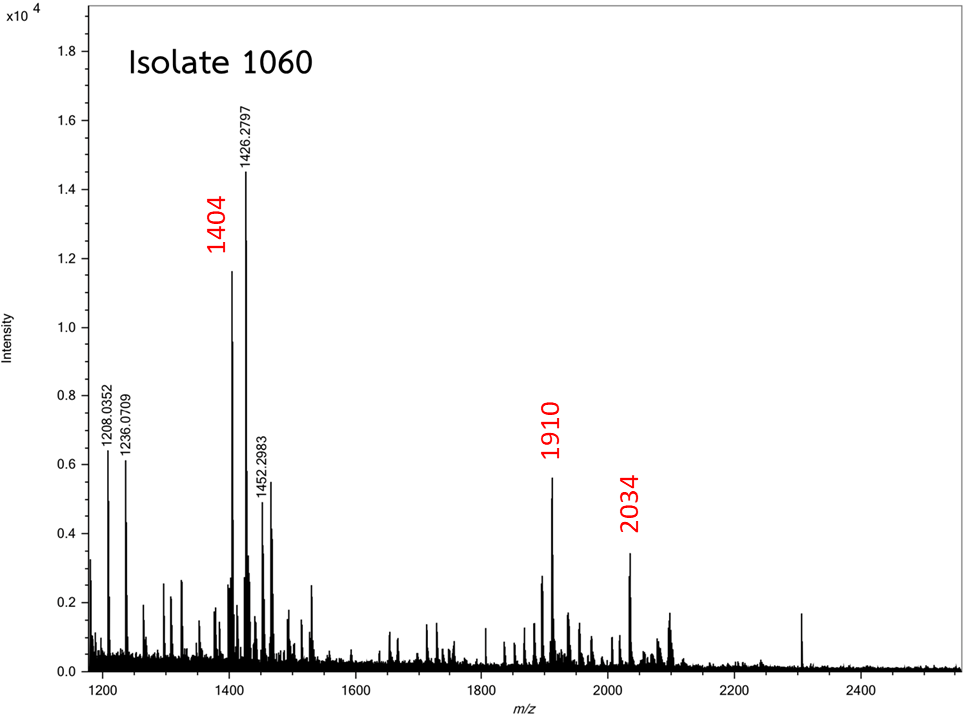

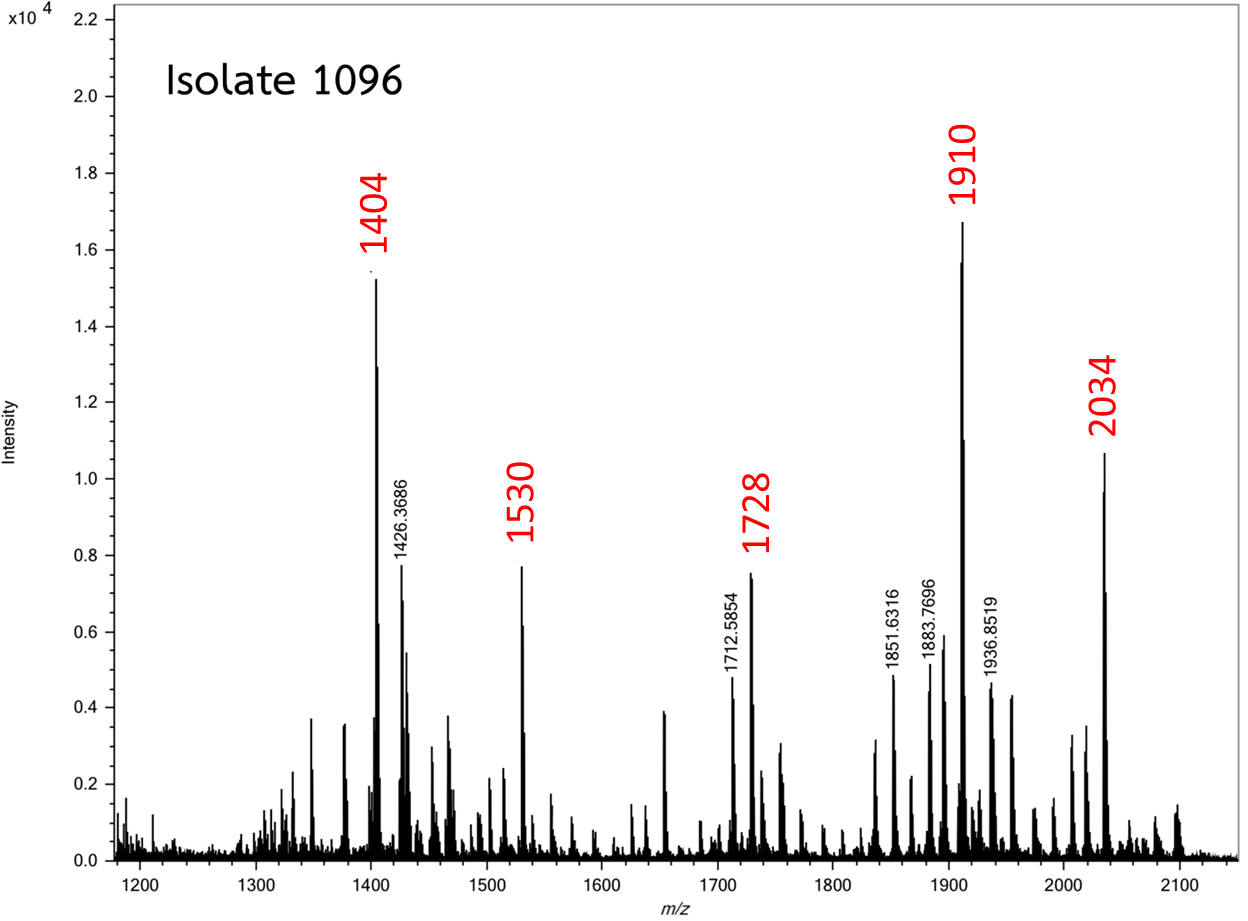

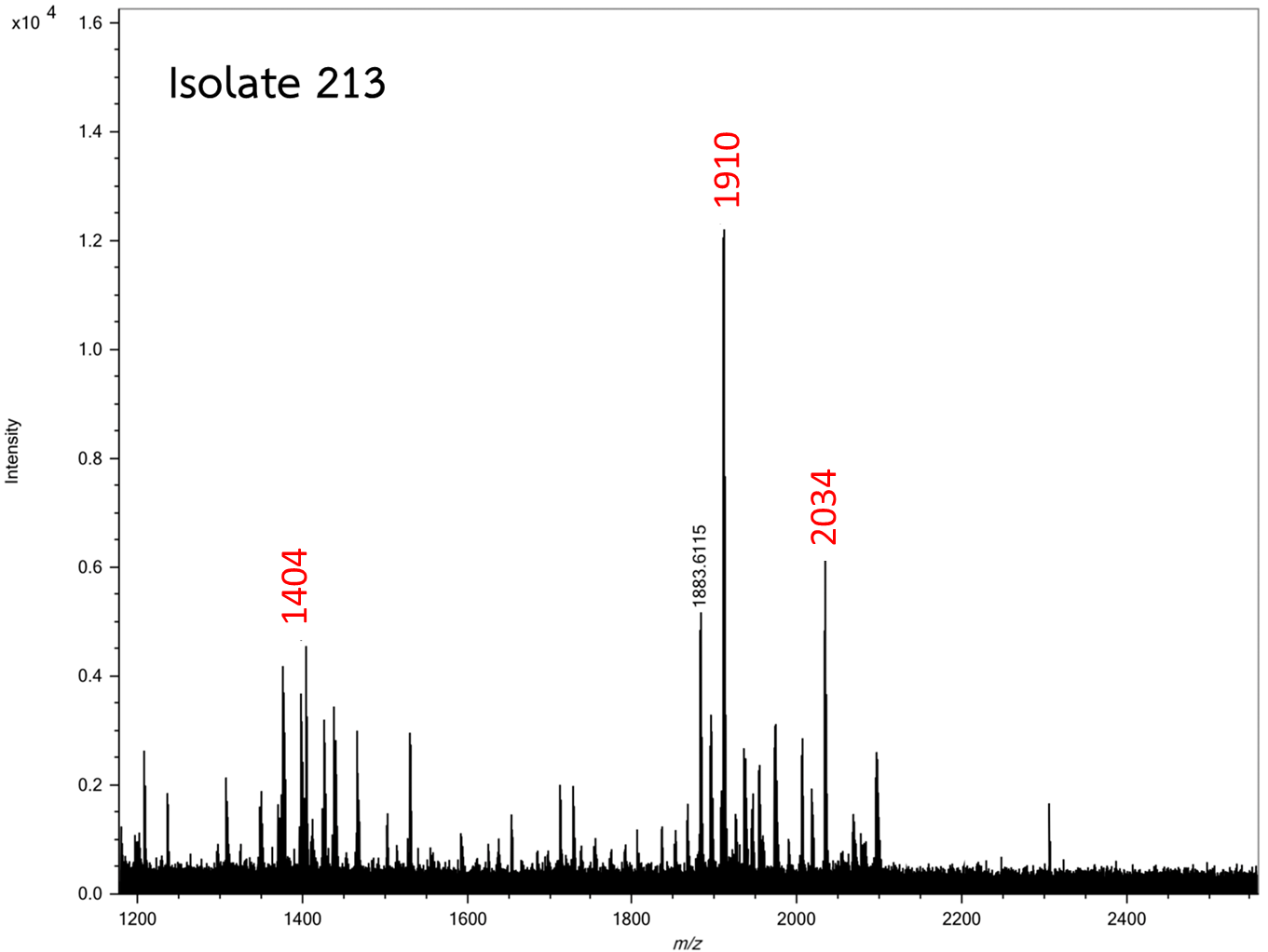

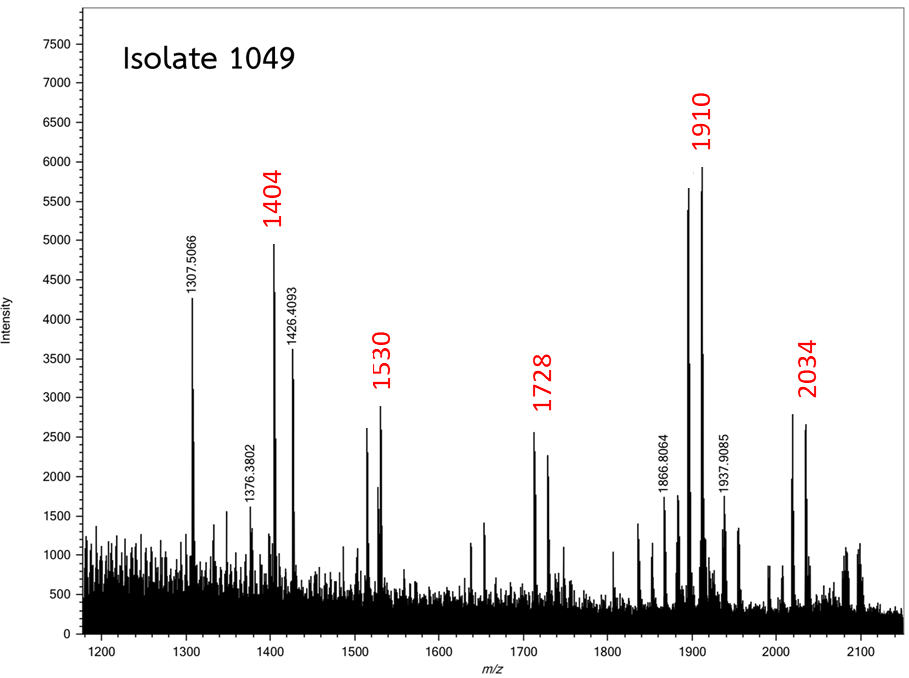
Figure 1 Lipid A spectra colistin-resistant *A. baumannii* clinical isolates which carrying phosphoethanolamine addition (n = 27).

Supplementary
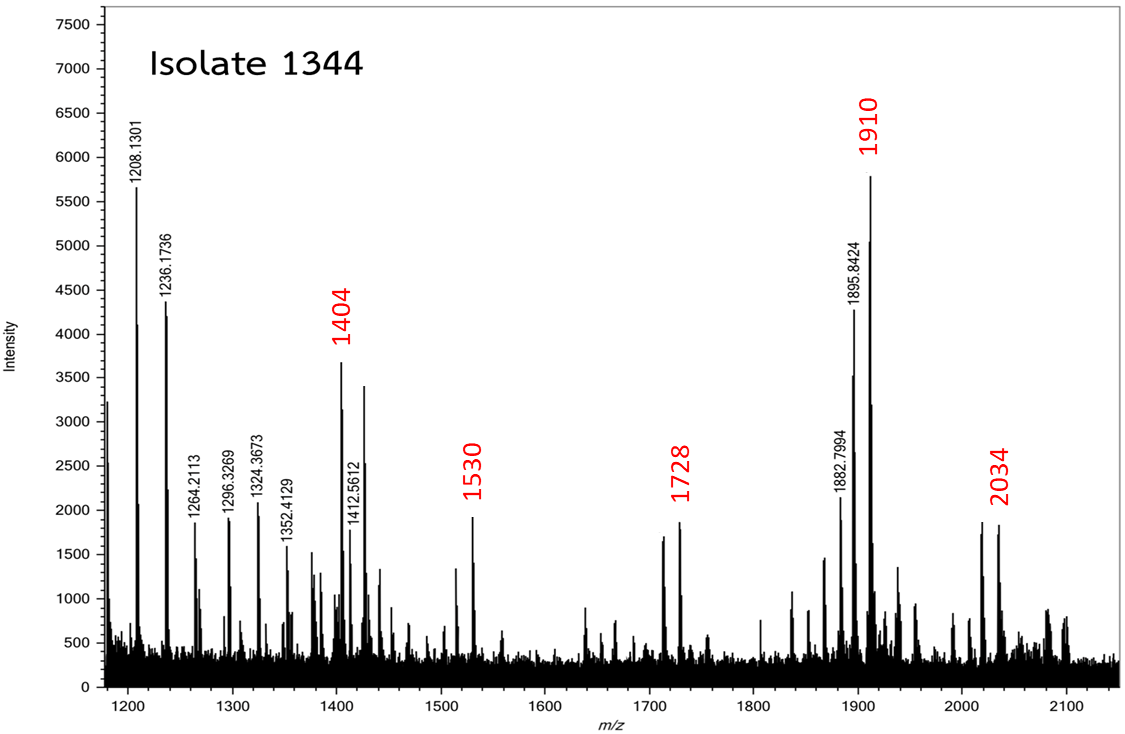

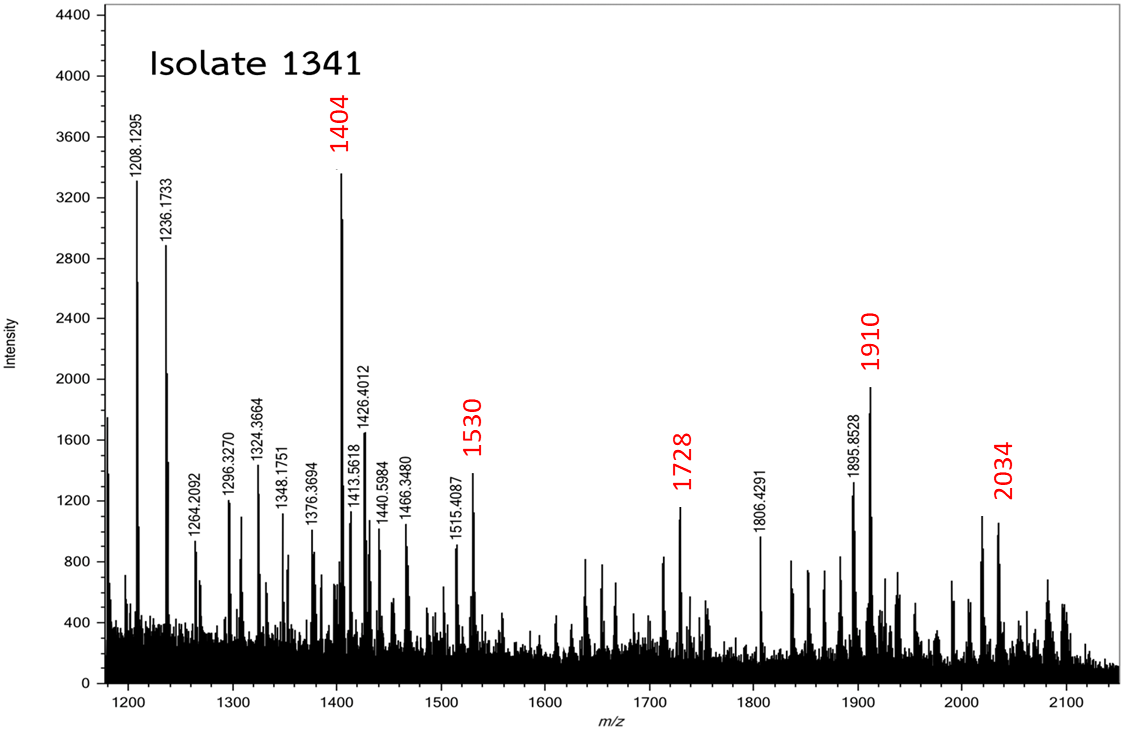

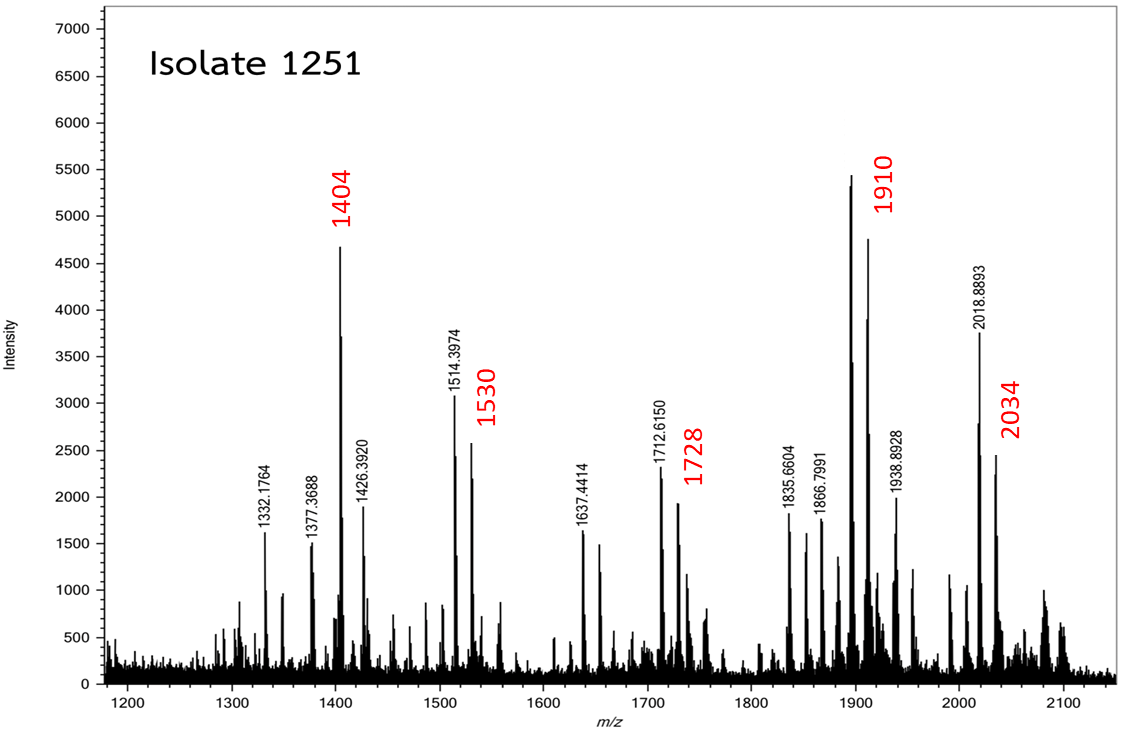

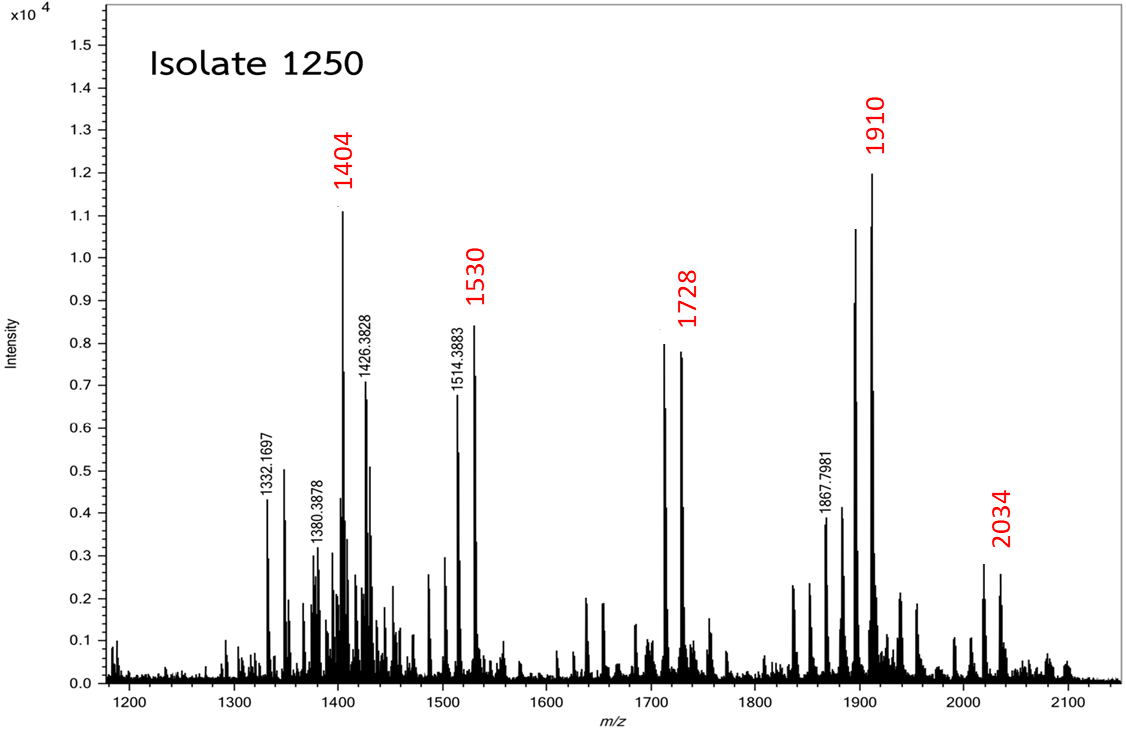

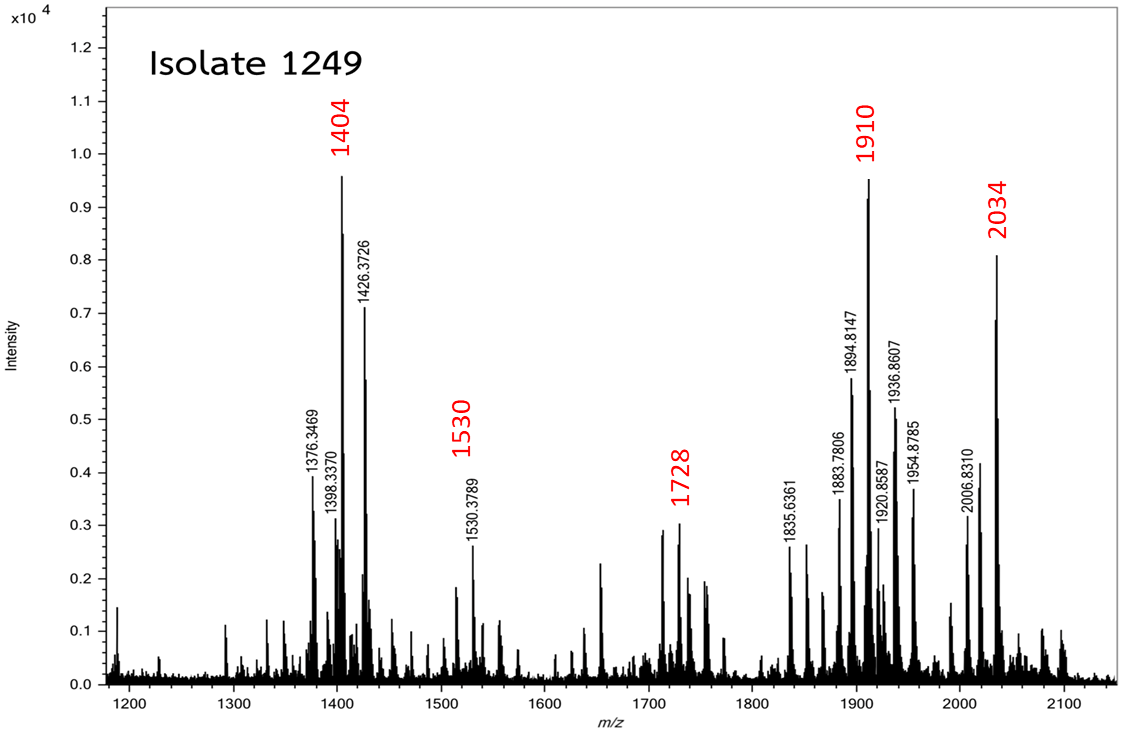

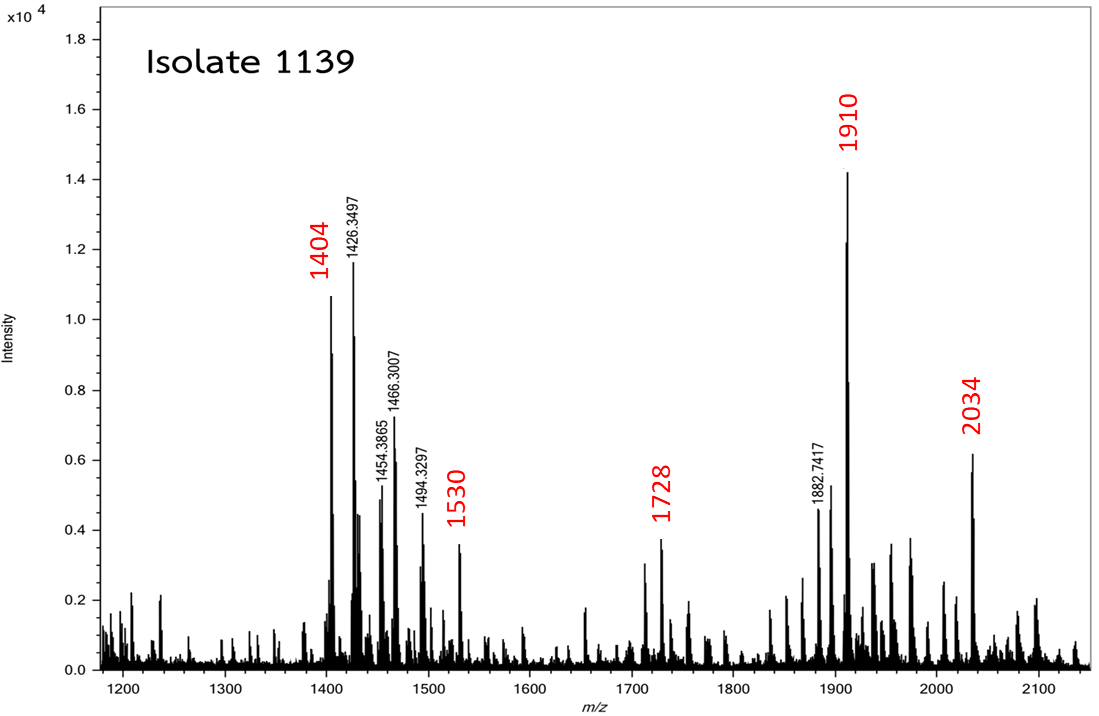
Figure 1 (continued)


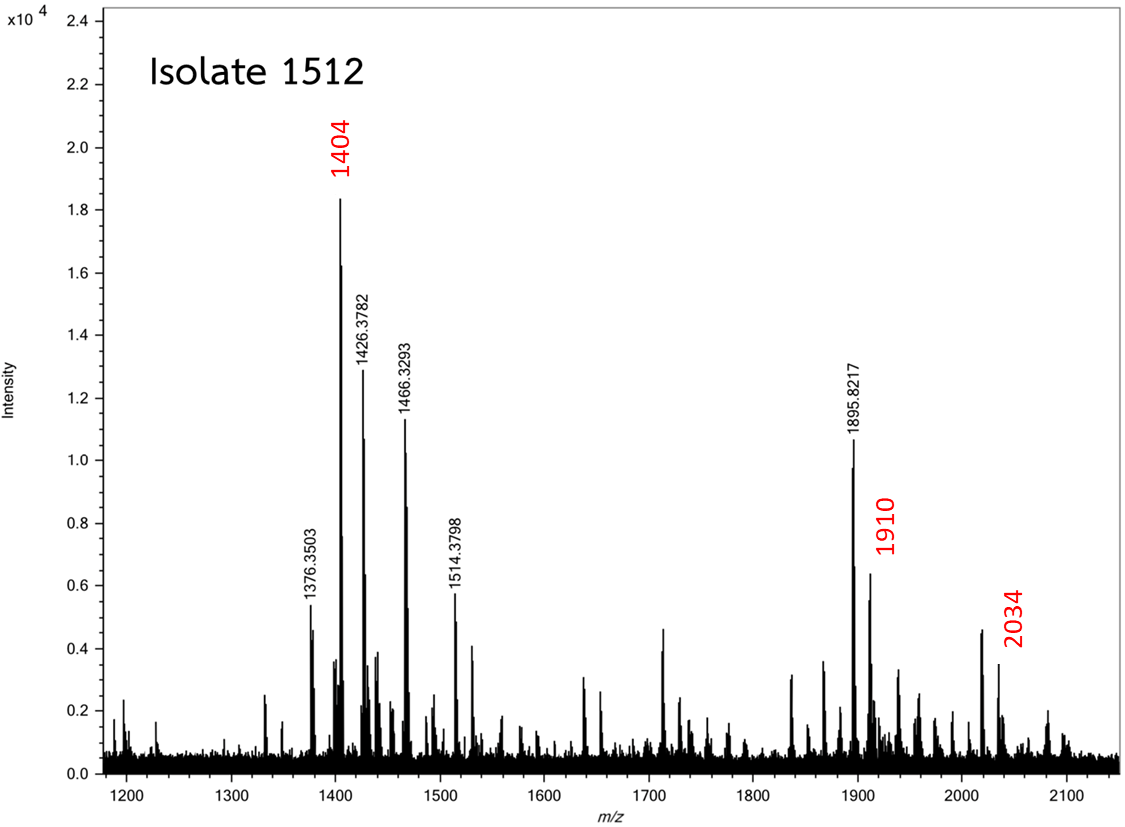

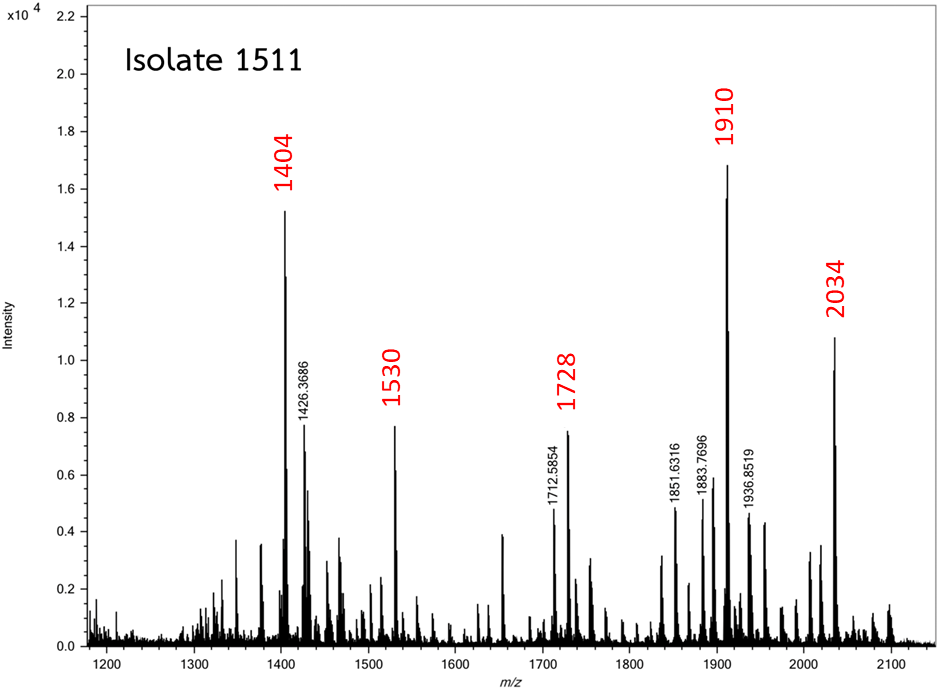

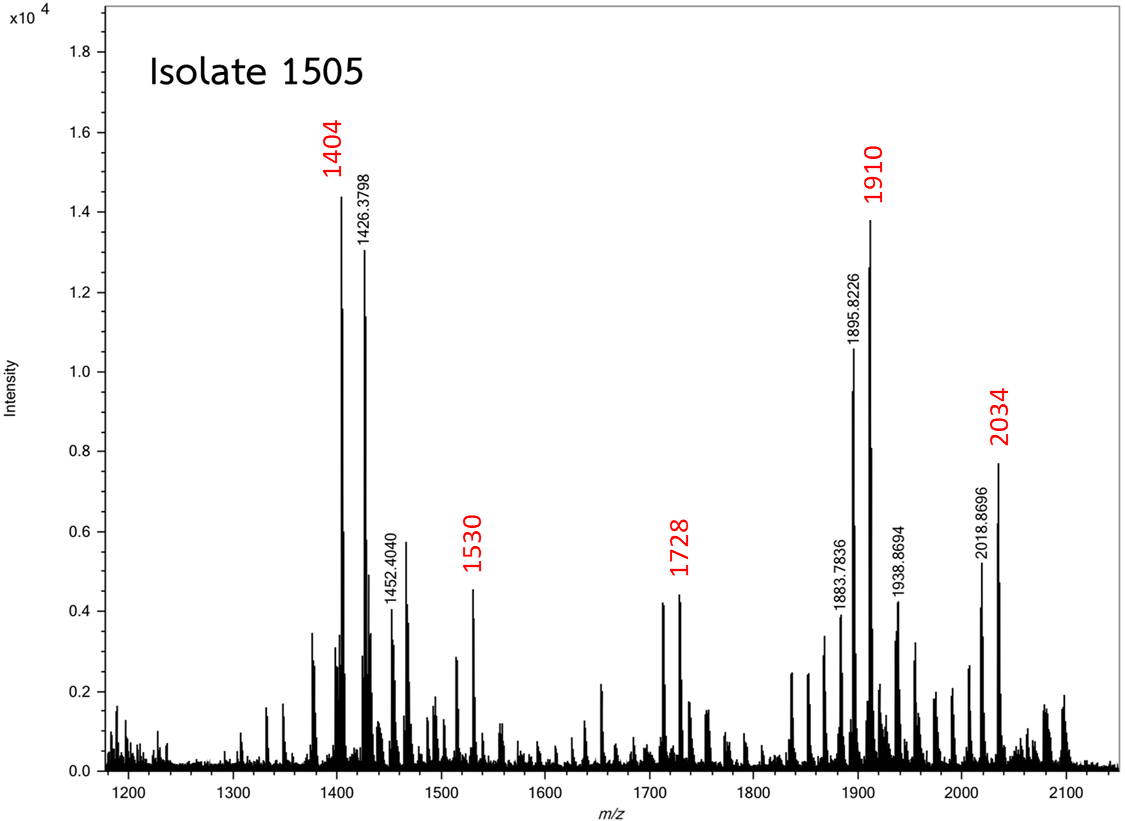

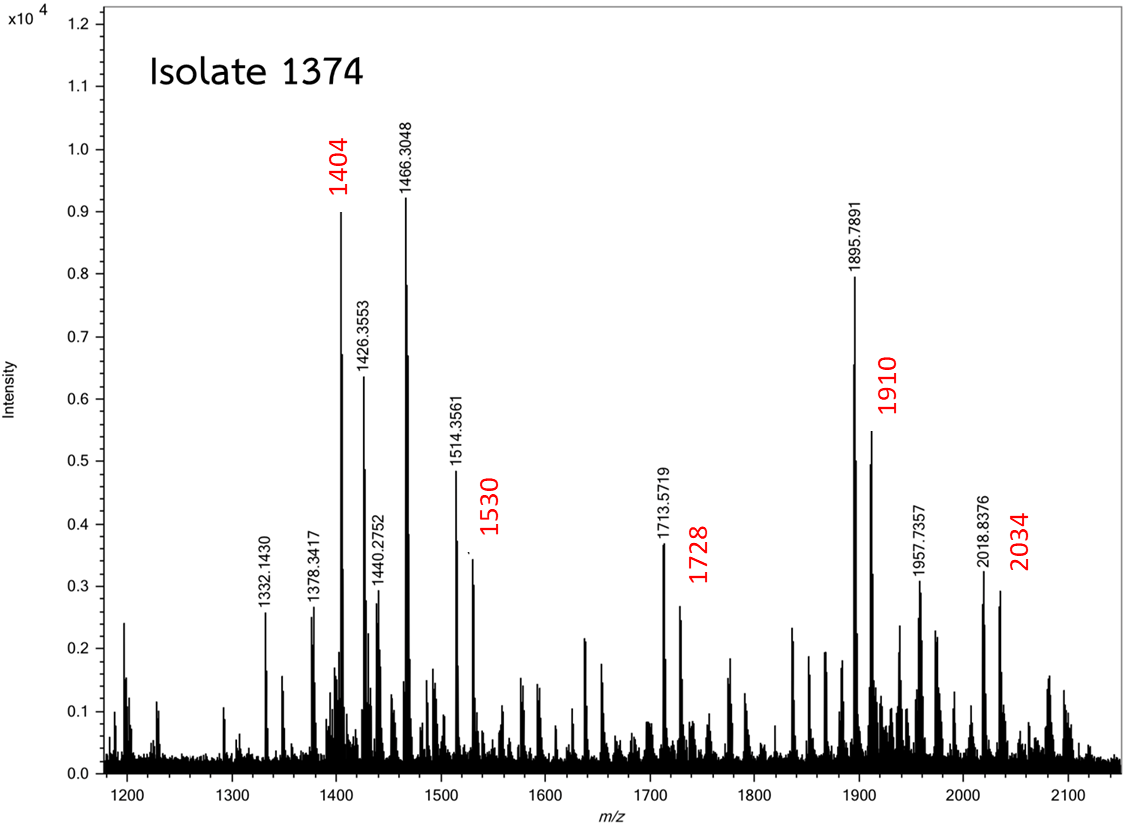

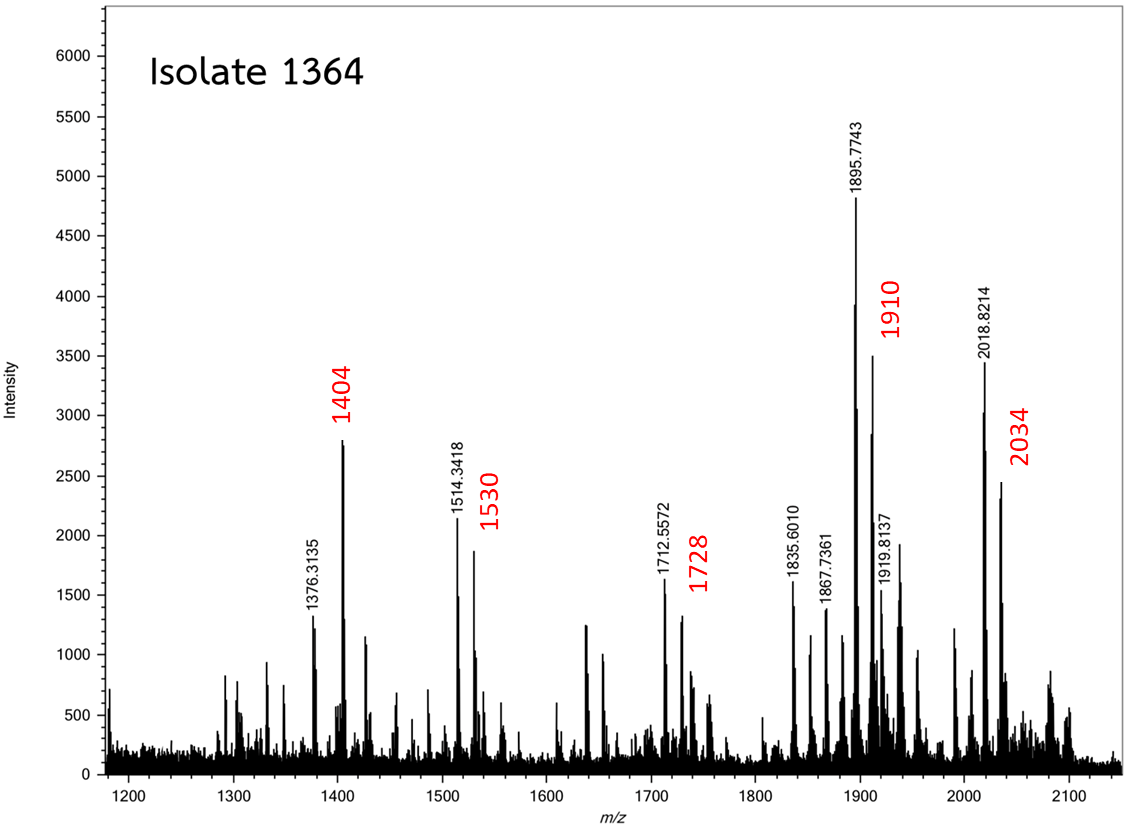

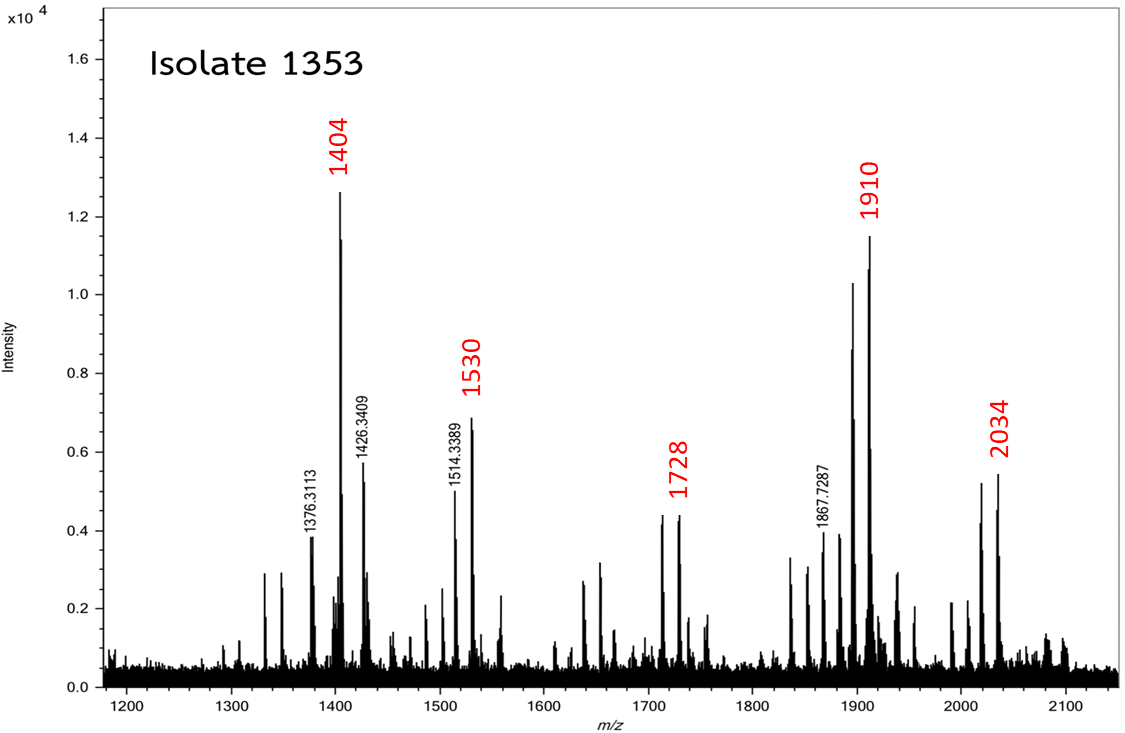
Figure 1 (continued)


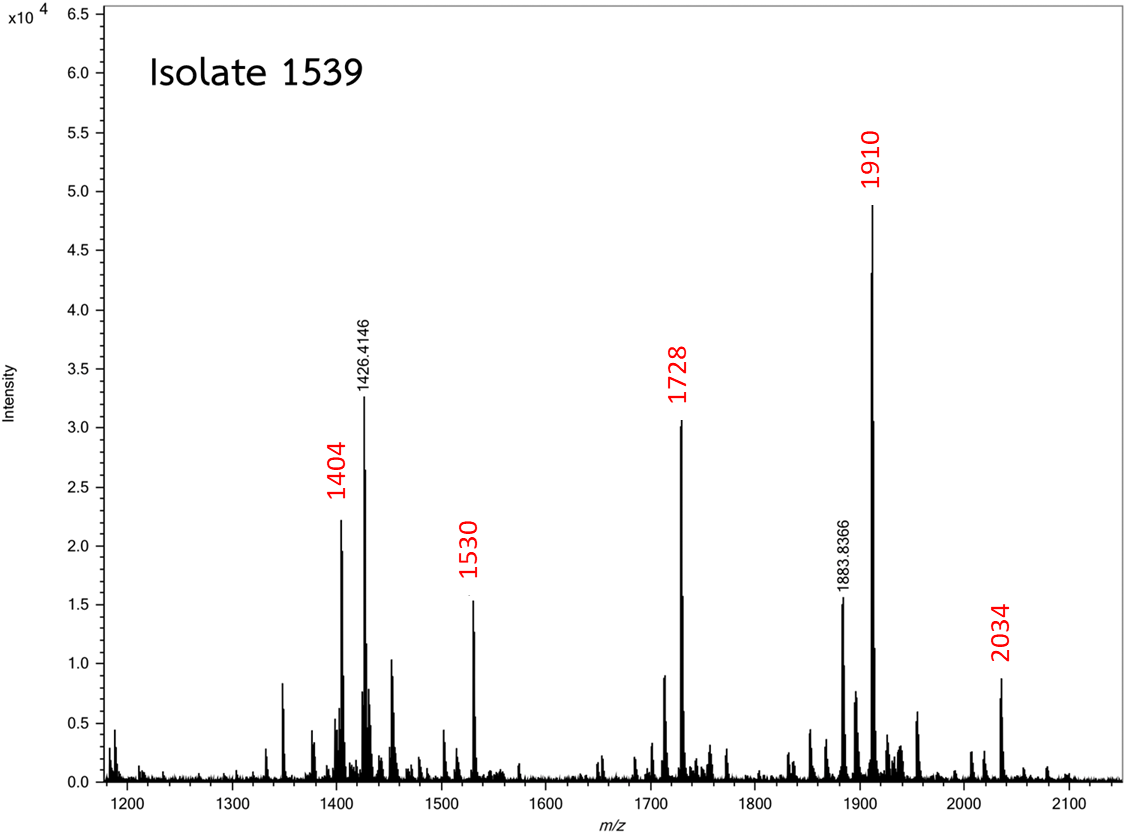

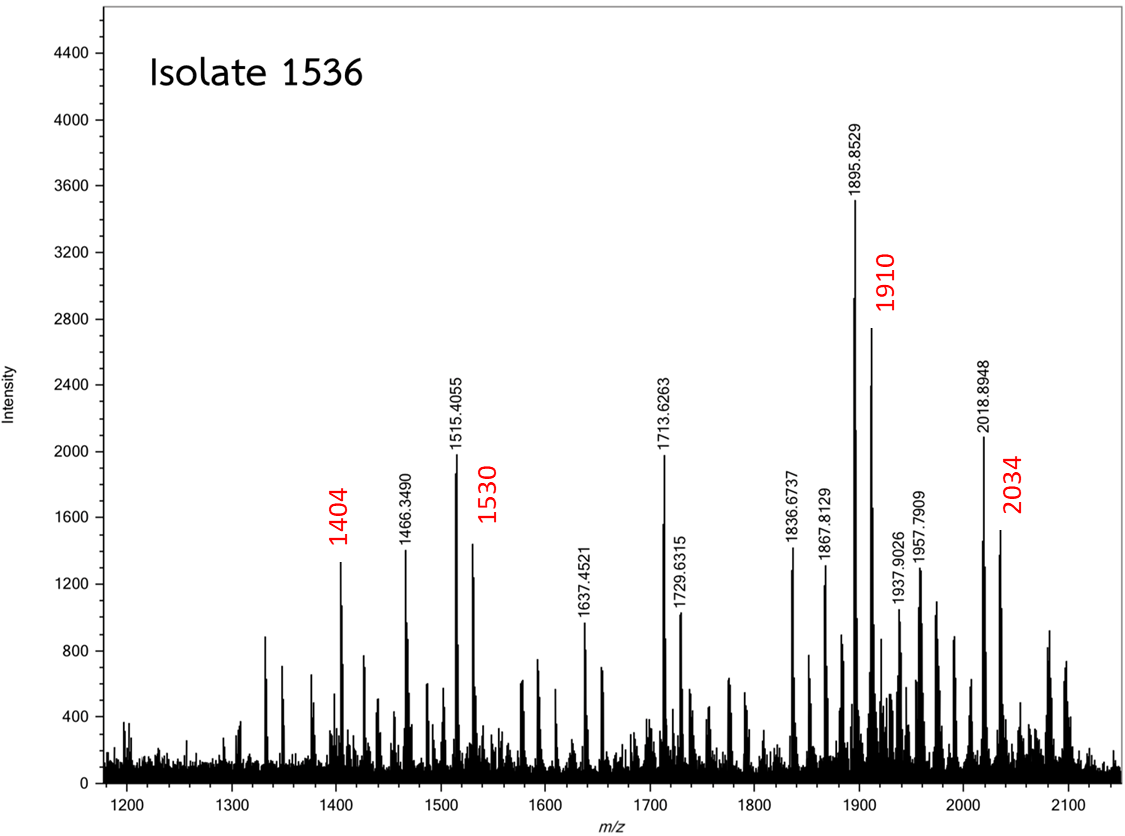

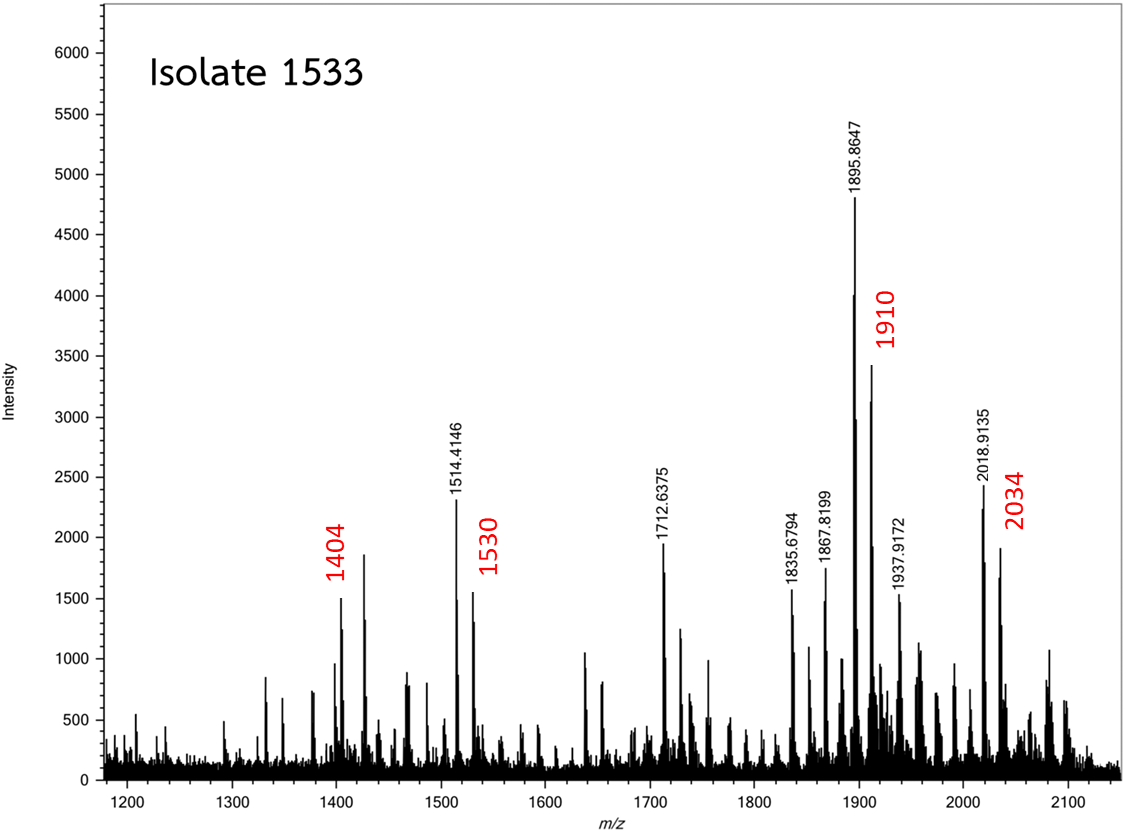

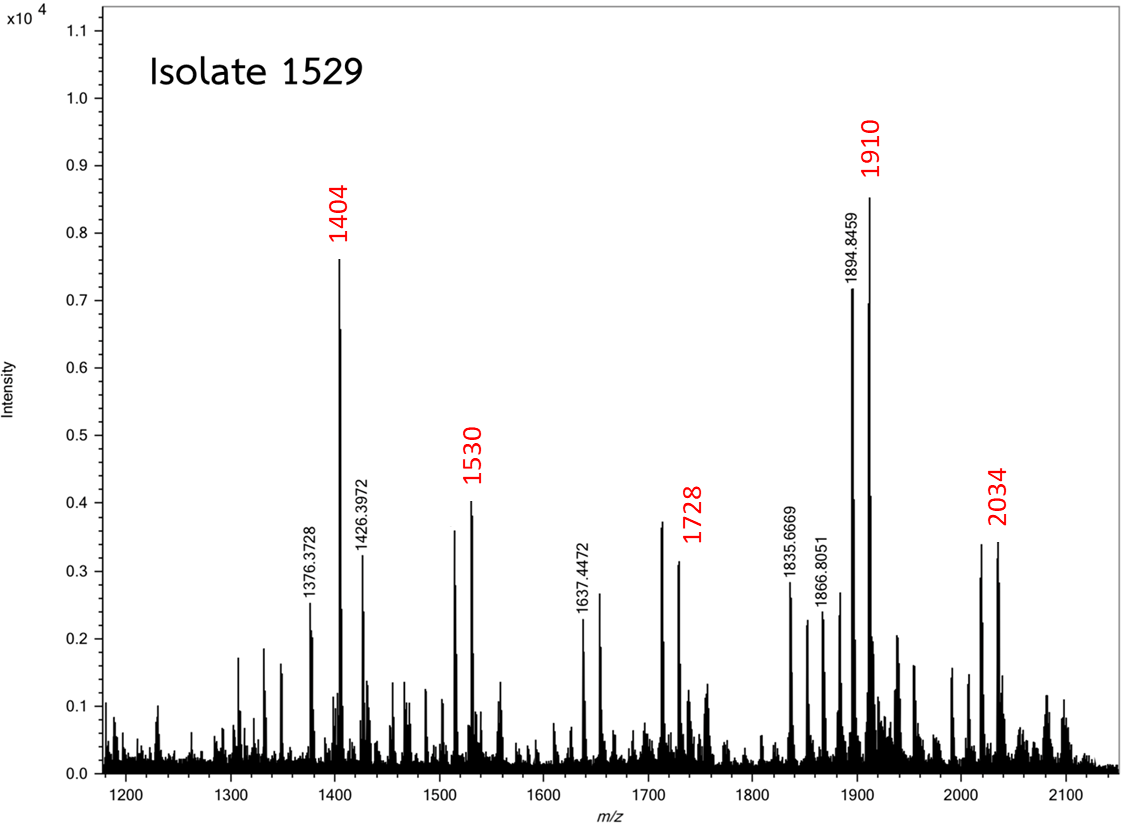

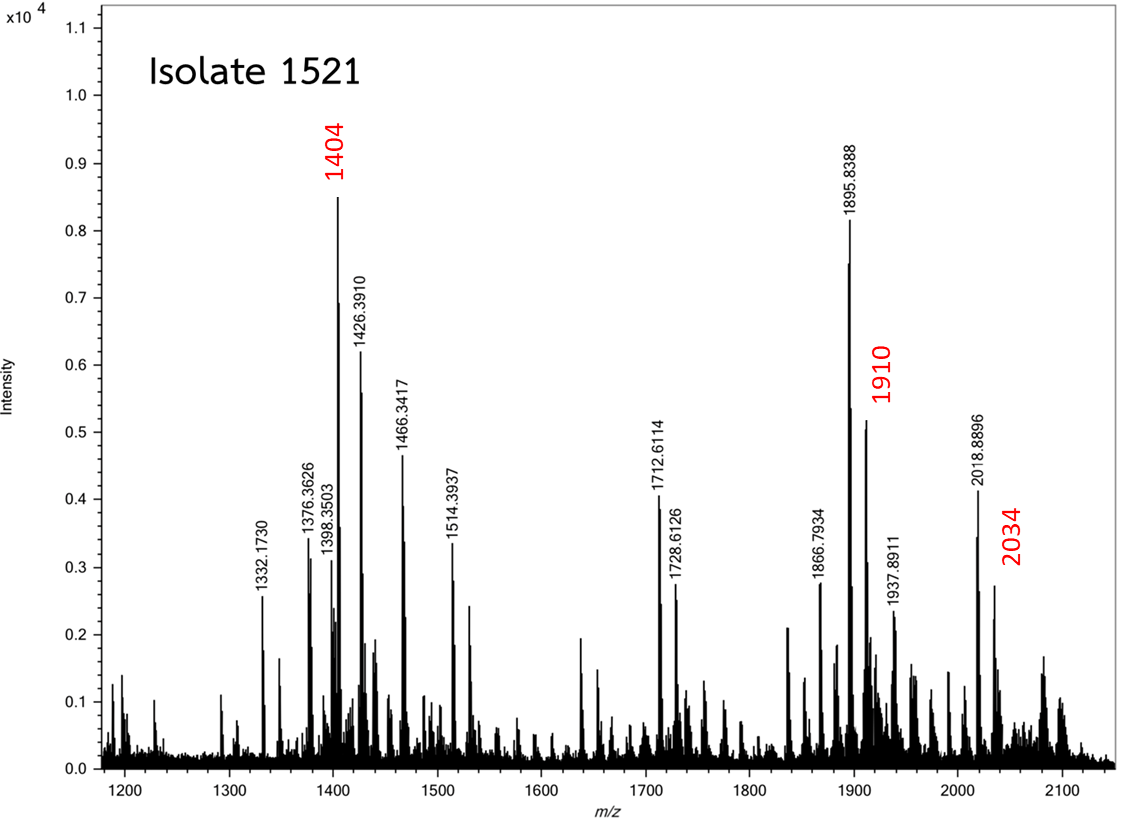

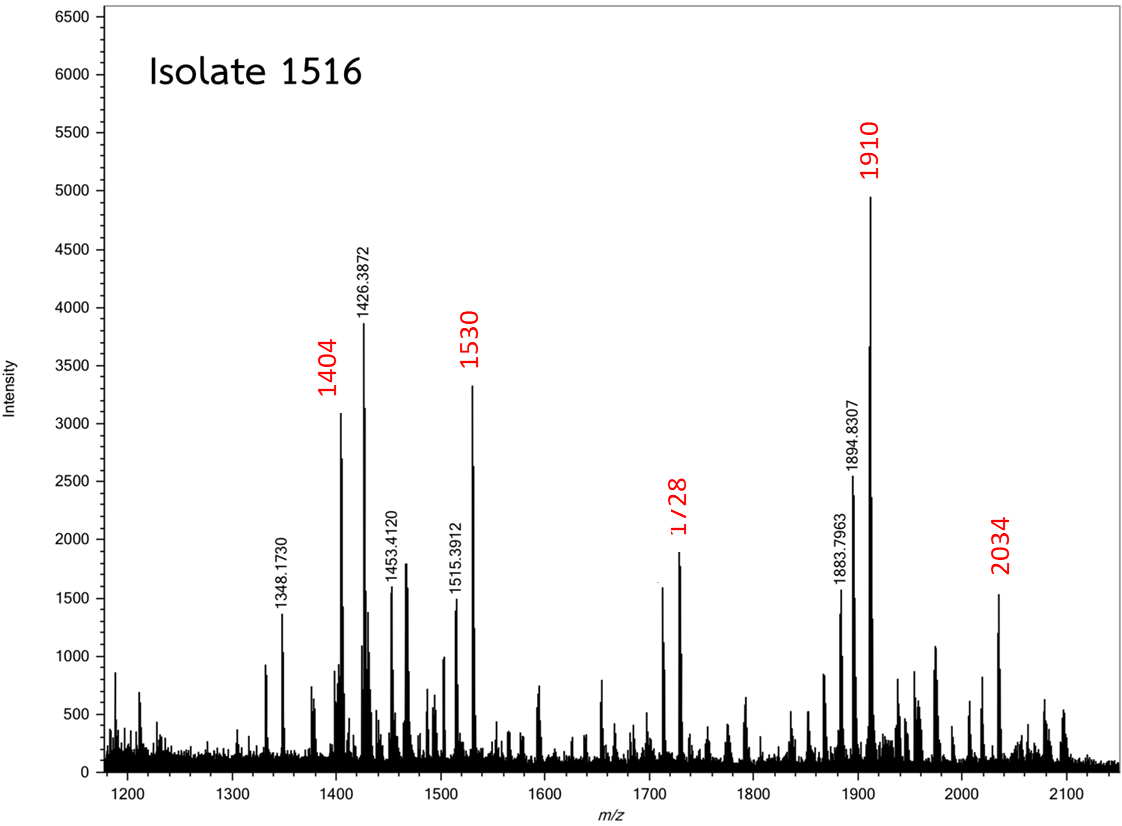
Figure 1 (continued)


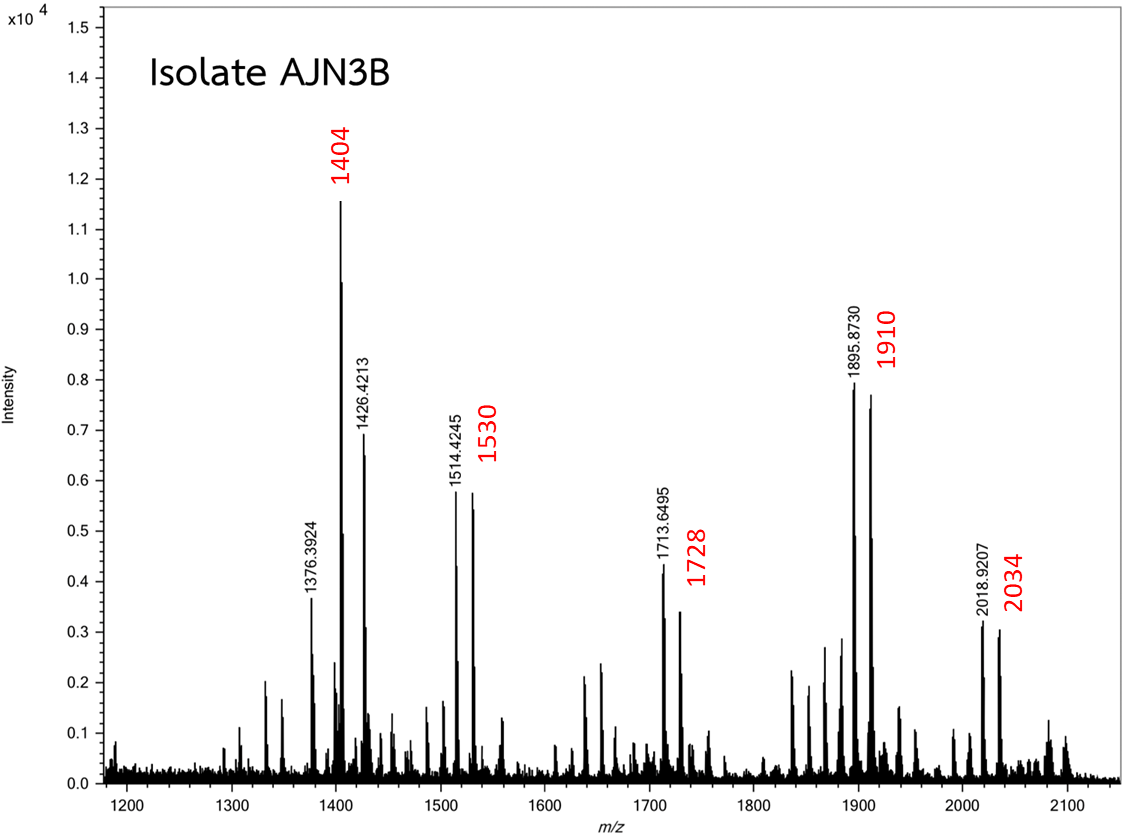

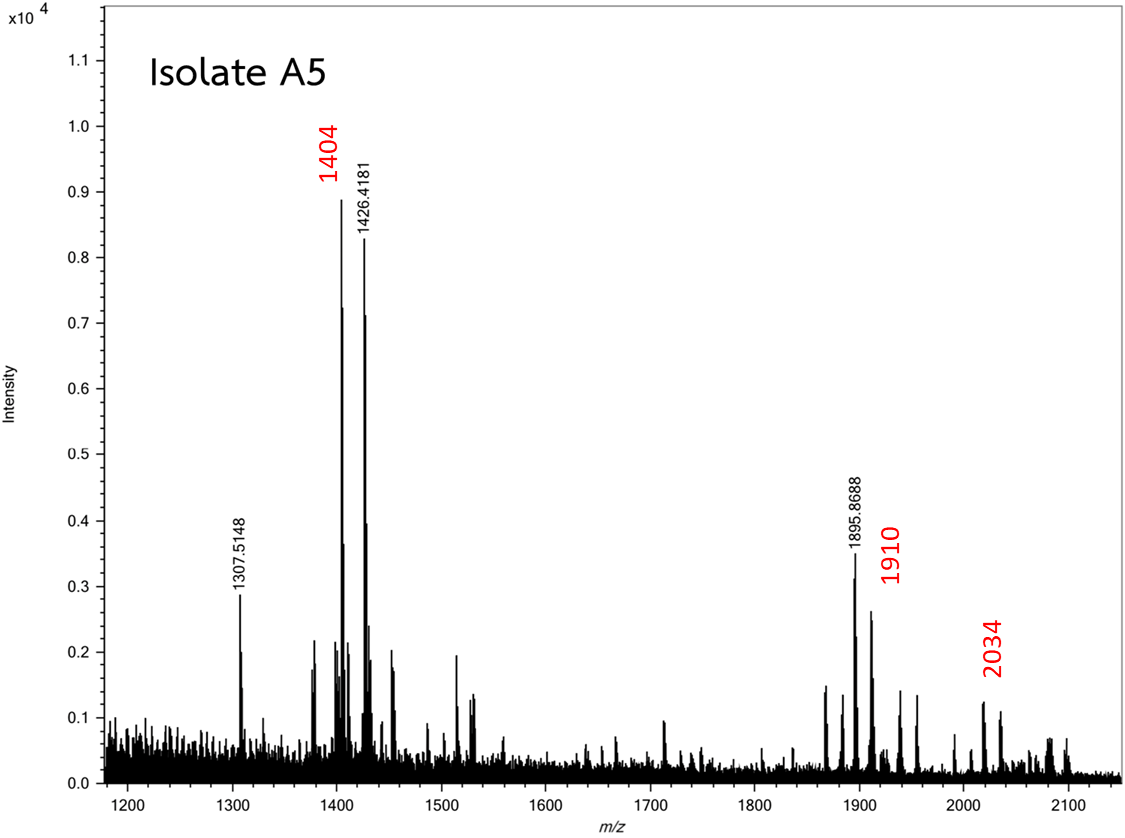

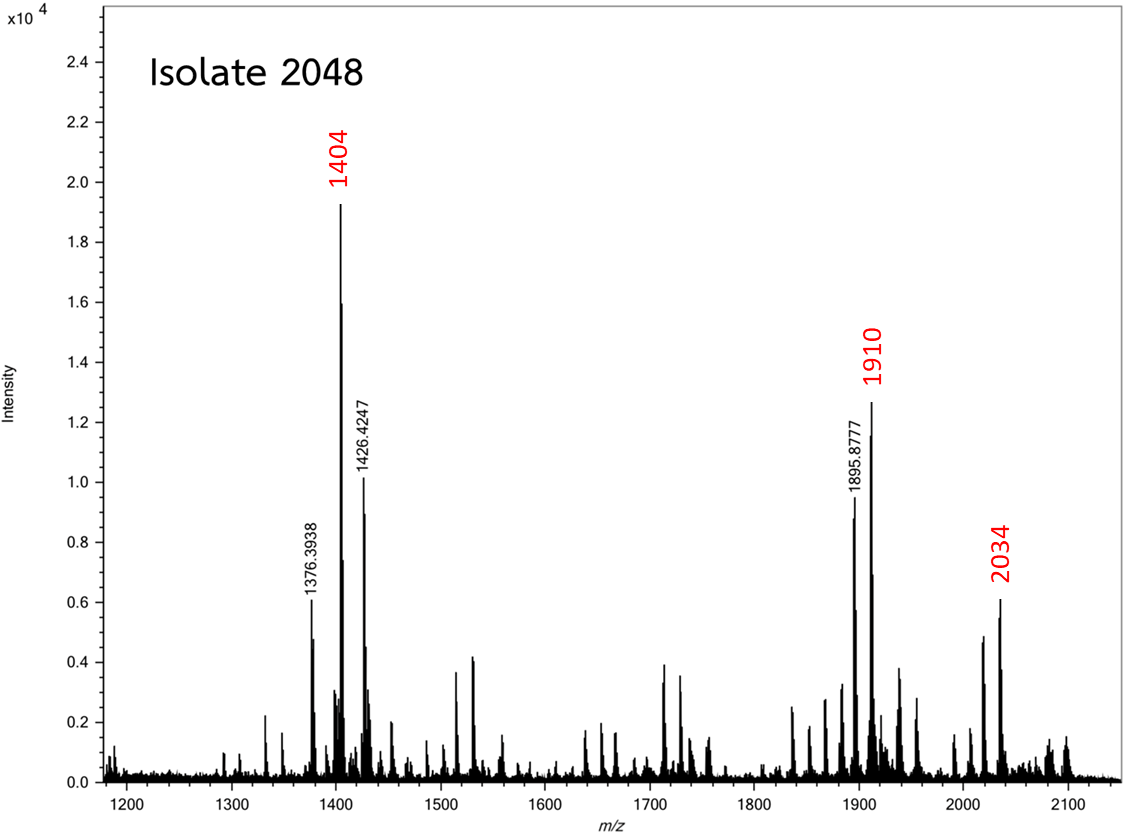
Figure 1 (continued)

Supplementary
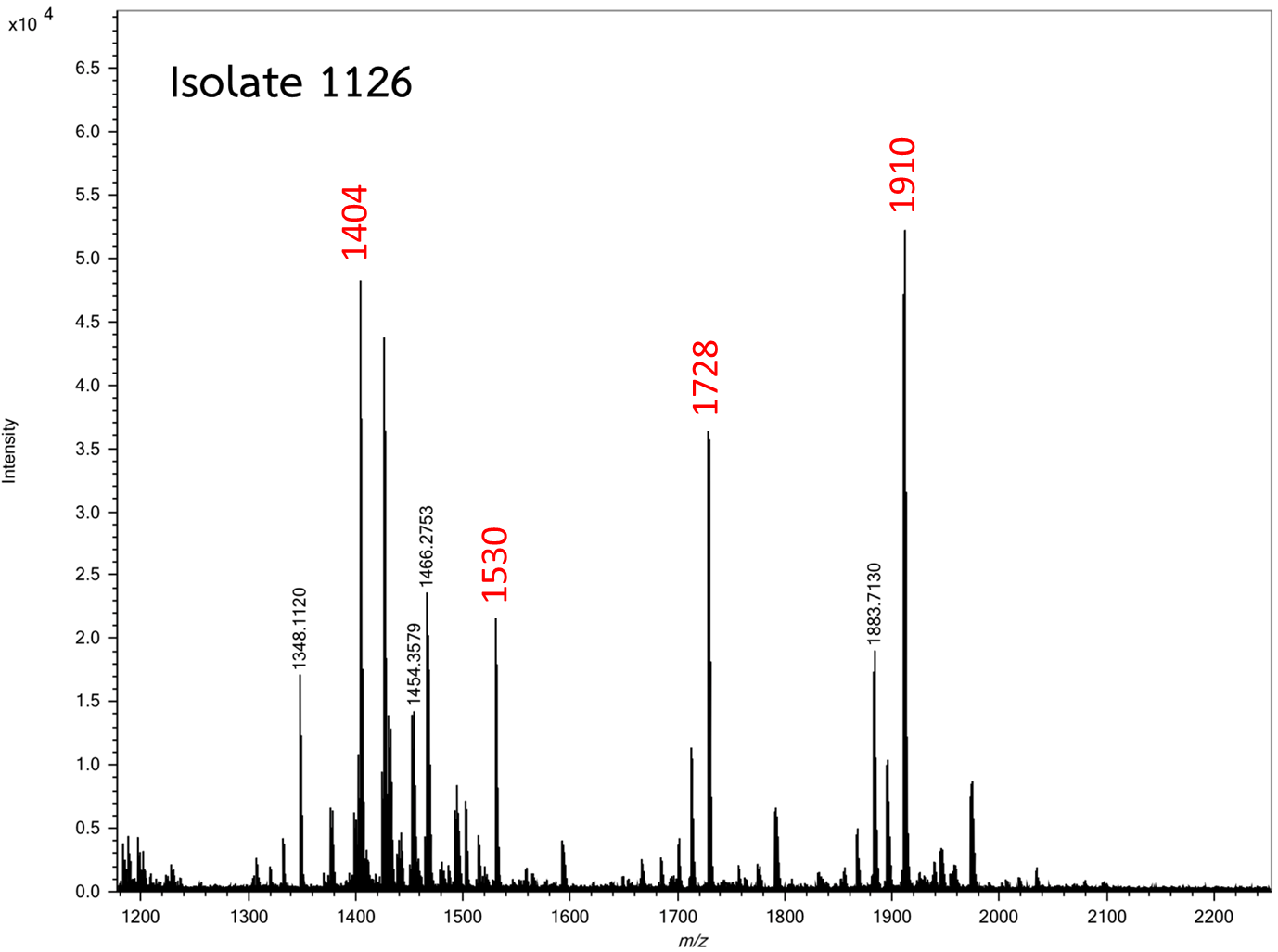

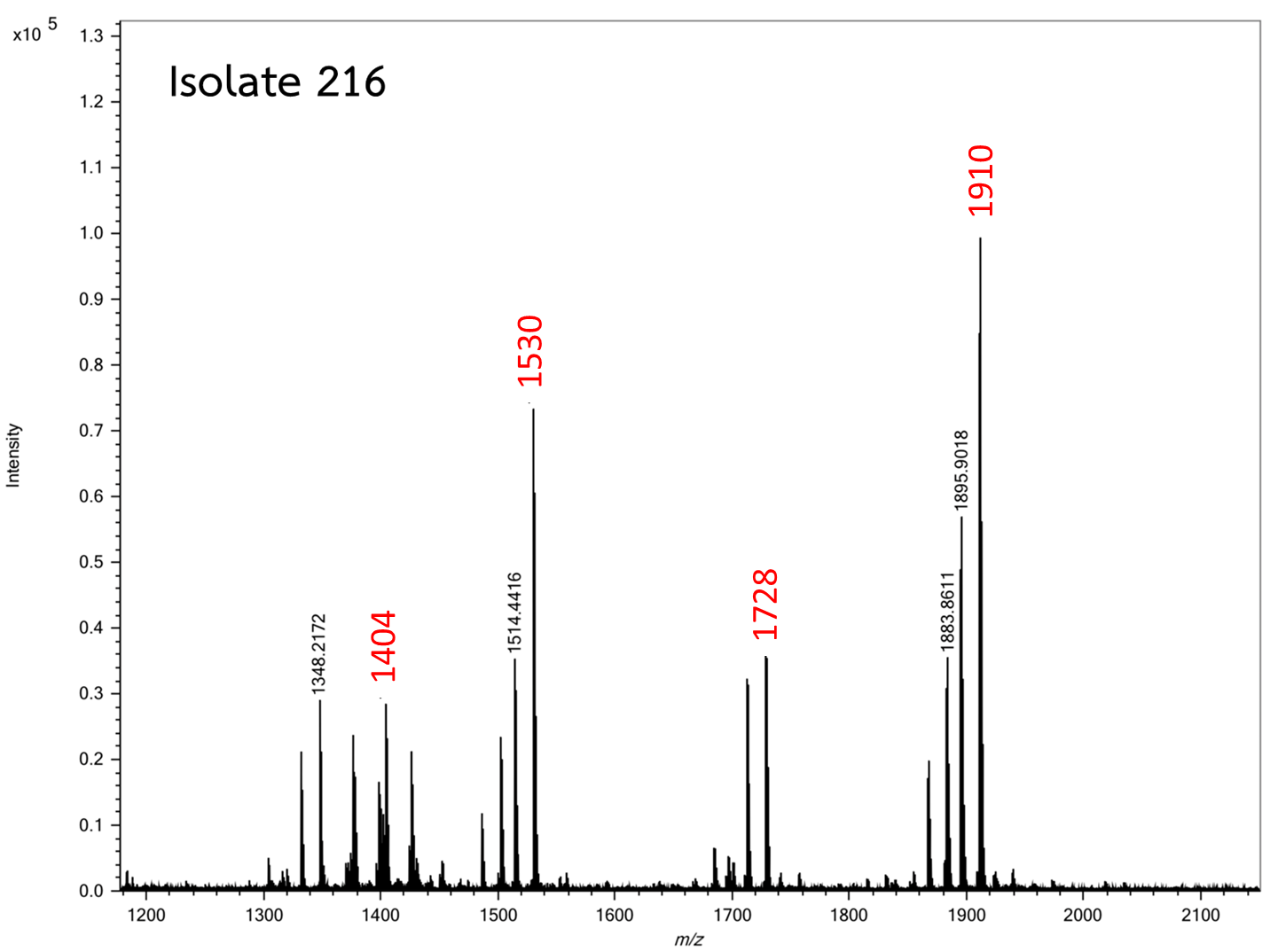

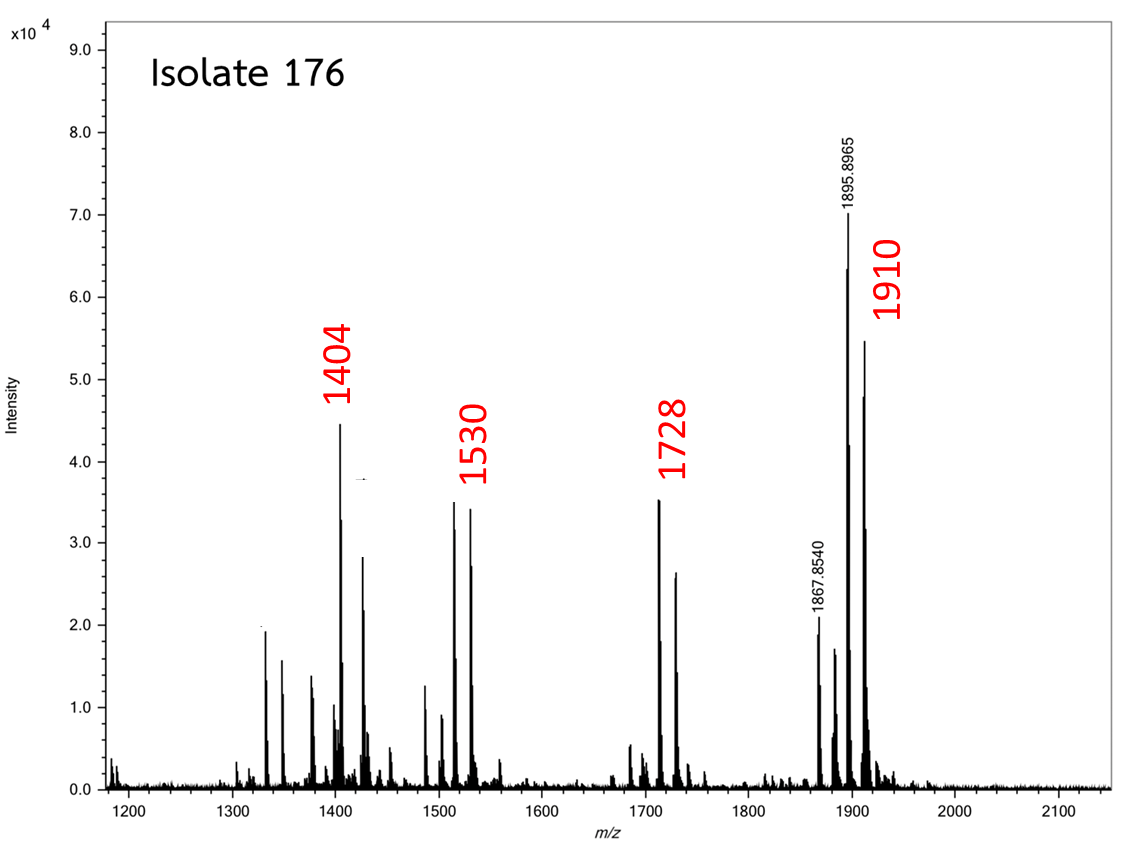
Figure 2 Lipid A spectra colistin-resistant *A. baumannii* clinical isolates which no phosphoethanolamine addition.

Supplementary Figure 3 Serum creatinine of mice


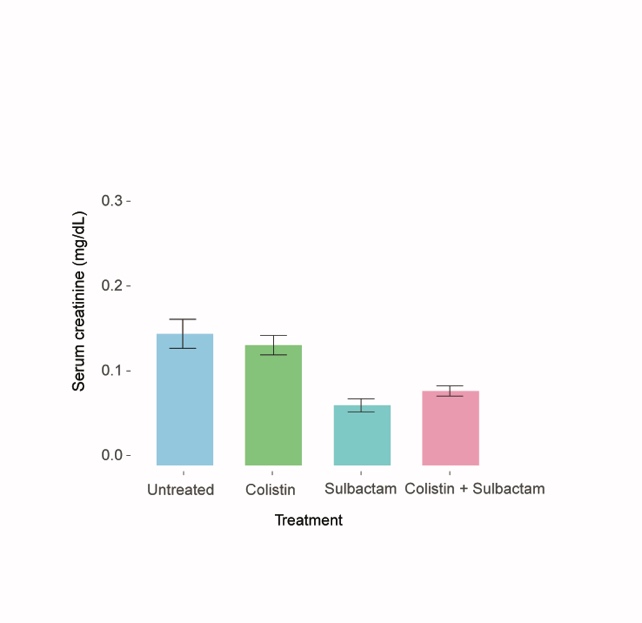


Supplementary Table 1. Fractional inhibitory concentration index (FICI) of colistin plus sulbactam, colistin plus fosfomycin, and sulbactam plus fosfomycin combinations against colistin-resistant *A. baumannii* clinical isolates (n = 30)

| Isolate | MIC (mg/L) | | | FICI*^a^* | | |
| --- | --- | --- | --- | --- | --- | --- |
|  | COL | SUL | FOS | COL + SUL | COL + FOS | SUL + FOS |
| 1529 | 4 | 16 | 256 | 0.5 | 0.31 | 0.38 |
| 1341 | 4 | 64 | 256 | 0.5 | 0.25 | 0.31 |
| 1511 | 4 | 16 | 512 | 0.5 | 1 | 0.38 |
| 216 | 4 | 32 | 512 | 0.5 | 0.75 | 0.75 |
| 1516 | 4 | 32 | 512 | 0.38 | 0.75 | 0.38 |
| 1536 | 4 | 32 | 512 | 0.38 | 1 | 0.5 |
| 176 | 4 | 128 | 512 | 0.5 | 0.75 | 0.75 |
| 1512 | 8 | 32 | 128 | 0.38 | 0.75 | 0.38 |
| 1049 | 8 | 16 | 256 | 0.5 | 0.5 | 0.63 |
| AJN3B | 8 | 16 | 256 | 0.53 | 0.75 | 0.75 |
| 1129 | 8 | 32 | 256 | 0.38 | 0.5 | 0.25 |
| 1353 | 8 | 32 | 256 | 0.25 | 1.5 | 0.38 |
| 1364 | 8 | 32 | 256 | 0.38 | 0.75 | 0.5 |
| 1505 | 8 | 32 | 256 | 0.25 | 0.75 | 0.25 |
| 1521 | 8 | 32 | 256 | 0.5 | 0.5 | 0.38 |
| 1539 | 8 | 32 | 256 | 0.38 | 0.75 | 0.25 |
| 2048 | 8 | 16 | 512 | 0.75 | 0.5 | 0.5 |
| 213 | 8 | 64 | 512 | 0.19 | 0.5 | 1 |
| 1251 | 16 | 32 | 128 | 0.25 | 0.38 | 0.5 |
| 1344 | 16 | 32 | 128 | 0.38 | 0.63 | 0.38 |
| A5 | 16 | 2 | 256 | 2 | 2 | 0.75 |
| 1249 | 16 | 32 | 256 | 0.25 | 0.63 | 0.38 |
| 1374 | 16 | 32 | 256 | 0.25 | 0.5 | 0.38 |
| 1098 | 16 | 32 | 512 | 0.38 | 0.75 | 0.38 |
| 1127 | 16 | 32 | 512 | 0.38 | 0.63 | 0.5 |
| 1533 | 16 | 32 | 512 | 0.38 | 0.75 | 0.5 |
| 1060 | 16 | 64 | 512 | 0.19 | 0.75 | 0.75 |
| 1126 | 32 | 16 | 512 | 0.19 | 0.5 | 0.75 |
| 1250 | 32 | 32 | 1024 | 0.38 | 0.63 | 0.38 |
| 1139 | 64 | 16 | 256 | 0.63 | 0.75 | 0.75 |

Supplementary Table 2 Primers for amplification of entire *pmrCAB* operon

| Primer name | Gene | Sequence (5´-3´) | Product size (bp) | reference |
| --- | --- | --- | --- | --- |
| FullpmrCAB-F | Entire *pmrCAB* | GCATCATAAAAAGATTGTAGTCAC | 3,699 | ^1^ |
| FullpmrCAB-R |  | GCGATTTGTATTCATCGTTTTGAG |  |  |
| pmrC-F |  | ATGTTTAATCTCATTATAGCCA |  |  |
| pmrC-R |  | TTAGTTTACATGGGCACAA |  |  |
| pmrC_2_-F |  | GGTTGTTATTGAAGAAAGTAT |  |  |
| pmrC_2_-R |  | TCAATCCAAGTCACTTGGTAAC |  |  |
| pmrA-F |  | ATGACAAAAATCTTGATGATTGAAGAT |  |  |
| pmrA-R |  | TTATGATTGCCCCAAACGGTAG |  |  |
| pmrB-F |  | GTGCATTATTCATTAAAAAAAC |  |  |
| pmrB-R |  | TCACGCTCTTGTTTCATGTA |  |  |
| pmrB_2_-F |  | GGTTCGTGAAGCTTTCG |  |  |
| pmrB_2_-R |  | CCTAAATCGATTTCTTTTTG |  |  |
| Dcap-F2 |  | AAACACCGACCACTGCAAAT |  | This study |
| pmrC-R2 |  | GCGTATGGTGCTCAGTTCTCT |  | This study |

Supplementary Table 3 Primers for amplification of *lpxA*, *lpxC*, *lpxD*

| Primer name | Gene | Sequence (5´-3´) | Product size (bp) | reference |
| --- | --- | --- | --- | --- |
| lpxC-F | *lpxC* | TGAAGATGACGTTCCTGCAA | 1,501 | ^2^ |
| lpxC-R |  | TGGTGAAAATCAGGCAATGA |  |  |
| lpxA-F | *lpxA* | TGAAGCATTAGCTCAAGTTT | 1,178 |  |
| lpxA-R |  | GTCAGCAAATCAATACAAGA |  |  |
| lpxD-F | *lpxD* | CAAAGTATGAATACAACTTTTGAG | 1,143 |  |
| lpxD-R |  | GTCAATGGCACATCTGCTAAT |  |  |

Supplementary Table 4 Primers for amplification of *mcr*-1 to *mcr*-9

| Primer name | Gene | Sequence (5´-3´) | Product size (bp) | reference |
| --- | --- | --- | --- | --- |
| mcr-1-F | *mcr-1* | AAAGACGCGGTACAAGCAAC | 213 | ^3^ |
| mcr-1-R |  | GCTGAACATGCACGGCACAG |  |  |
| mcr-2-F | *mcr-2* | CGACCAAGCCGAGTCTAAGG | 92 |  |
| mcr-2-R |  | CAACTGCGACCAACACACTT |  |  |
| mcr-3-F | *mcr-3* | ACCTCCAGCGTGAGATTGTTCCA | 169 |  |
| mcr-3-R |  | GCGGTTTCACCAACGACCAGAA |  |  |
| mcr-4-F | *mcr-4* | AGAATGCCACTCGTAACCCG | 230 |  |
| mcr-4-R |  | GCGAGGATCATAGTCTGCCC |  |  |
| mcr-5-F | *mcr-5* | CTGTGGCCAGTCATGGATGT | 98 |  |
| mcr-5-R |  | CGAATGCCCGAGATGACGTA |  |  |
| mcr-6-F | *mcr-6* | AGCTATGTCAATCCCGTGAT | 252 | ^4^ |
| mcr-6-R |  | ATTGGCTAGGTTGTCAATC |  |  |
| mcr-7-F | *mcr-7* | GCCCTTCTTTTCGTTGTT | 551 |  |
| mcr-7-R |  | GGTTGGTCTCTTTCTCGT |  |  |
| mcr-8-F | *mcr-8* | TCAACAATTCTACAAAGCGTG | 856 |  |
| mcr-8-R |  | AATGCTGCGCGAATGAAG |  |  |
| mcr-9-F | *mcr-9* | TTCCCTTTGTTCTGGTTG | 1011 |  |
| mcr-9-R |  | GCAGGTAATAAGTCGGTC |  |  |

REFERENCE

1 Beceiro, A. *et al.* Phosphoethanolamine modification of lipid A in colistin-resistant variants of *Acinetobacter baumannii* mediated by the PmrAB two-component regulatory system. *Antimicrob Agents Chemother* **55**, 3370-3379, doi:10.1128/AAC.00079-11 (2011).

2 Moffatt, J. H. *et al.* Colistin resistance in *Acinetobacter baumannii* is mediated by complete loss of lipopolysaccharide production. *Antimicrob Agents Chemother* **54**, 4971-4977, doi:10.1128/AAC.00834-10 (2010).

3 Tolosi, R. *et al.* Rapid detection and quantification of plasmid-mediated colistin resistance genes (*mcr-1* to *mcr-5*) by real-time PCR in bacterial and environmental samples. *J Appl Microbiol* **129**, 1523-1529, doi:10.1111/jam.14738 (2020).

4 Borowiak, M. *et al.* Identification of a novel transposon-associated phosphoethanolamine transferase gene, *mcr*-5, conferring colistin resistance in d-tartrate fermenting *Salmonella enterica* subsp. *enterica* serovar Paratyphi B. *J Antimicrob Chemother* **72**, 3317-3324, doi:10.1093/jac/dkx327 (2017).
